# Reaching and engaging people: Analyzing tweeting practices of large U.S. police departments pre- and post- the killing of George Floyd

**DOI:** 10.1371/journal.pone.0269288

**Published:** 2022-07-14

**Authors:** Beidi Dong, Xiaoyun Wu

**Affiliations:** 1 Department of Criminology, Law and Society, George Mason University, Fairfax, VA, United States of America; 2 National Policing Institute, Arlington, VA, United States of America; Universiti Pertahanan Nasional Malaysia, MALAYSIA

## Abstract

Finding ways to improve police legitimacy and police-community relations has for long been an important social issue in the United States. It becomes particularly urgent following the murder of George Floyd on May 25^th^, 2020. An emerging area that holds potential in remediating police-community relations pertains to the use of social media by police. Yet, this body of research stays highly exploratory (e.g., case studies based on a small sample of agencies) and different viewpoints exist regarding the objectives of police social media usage. The current study identified 115 large police departments in the U.S. and collected their tweets over a 4-month period between 4/1/2020 and 7/31/2020. We investigated how police agencies (both individually and as an aggregate) leveraged social media to respond to the nationwide protests directed at the police and community reactions to such responses. We found that police agencies tweeted more frequently in the immediate aftermath of the murder and posted an increased number of civil-unrest related tweets. The public showed a greater interest in engaging with law enforcement agencies (i.e., average favorite and retweet counts) following the murder. A great variability emerged across agencies in their responses on social media, suggesting that examining only a handful of agencies or a particular dimension of social media usage would limit our understanding of police behaviors and citizen interactions on social media. In conclusion, we suggested a few avenues for future research (and practices) on responsible and effective use of social media by police, while pointing out the challenges associated with such inquiries.

## Introduction

On May 25, 2020, George Floyd, a 46-year-old Black man arrested on suspicion of using a counterfeit $20 bill, was murdered by Derek Chauvin, a 44-year-old white police officer in the Minneapolis Police Department who knelt on Floyd’s neck for almost 10 minutes. Following the killing of George Floyd, the social protest movement across the United States (U.S.) in the summer of 2020 led to a new round of contentious debates on police work, with many calling for fundamental police reform (e.g., to “defund the police”) and enhanced accountability. Inquiries on ways to improve police legitimacy and police-community relations have not been more urgent, as law enforcement agencies continue to represent the primary source of social control and the public rely on them for protection in a turbulent social environment (e.g., the recent spike of homicides amidst an ongoing pandemic) [1–5].

An emerging area that holds potential in remediating police-community relations pertains to the use of social media by police [6]. Throughout the protests, we saw police departments across jurisdictions engaging in public conversations and other activities on social media, some of which garnered extensive public attention. Despite some preliminary evidence suggesting the potential benefits of a police presence on social media, research on police use of social media has been relatively scant, as well as mixed [7–10]. Advocates for increasing police presence on social media suggested a myriad of potential benefits, including improving community outreach, investigation, and crime prevention [11, 12]. Inversely, police social media usage was argued to mainly fulfill a function of socialization (i.e., people internalize how police think and what police value) or legitimation (i.e., police justify contested actions through direct information sharing), thereby mediating public pressure for reform [13]. Additionally, there are concerns that police mostly engage in shallow, non-dialogical interactions with the public on social media [14–16].

In light of these dissimilar viewpoints of police social media usage, the current study seeks to understand how police in the U.S. leveraged social media to respond to the killing of George Floyd and to the nationwide protests that ensued. The study is motivated by 1) an increasing social media presence of law enforcement agencies [17], 2) ongoing frictions between police and disadvantaged and minority communities, 3) perceived benefits and challenges of social media usage among police practitioners, and 4) limited research covering police use of social media and its impact across the nation. The scale and intensity of the protest, amidst a global pandemic that ushered in a period of rapid growth in digital communication generally [18] and in policing [19], provide us with an exceptional opportunity to examine police social media usage and community reactions to it.

### Police use of social media

Police presence on social media has become growingly prevalent in the U.S. and other countries over the past decade. In a recent law enforcement use of social media survey in the U.S., Kim and colleagues noted that about 96% of their agency respondents (N = 539) affiliated with the International Association of Chiefs of Police (IACP) have a social media account as of 2016, with most agencies adopting social media usage between 2010 and 2014 [17]. Social media is thought to also serve as a technological driver of open government initiatives. The Open Government Directive of the Obama administration propels government agencies to provide more information to the public and to establish mechanisms through which public feedback can be collected and used to evaluate and improve government performance [9, 20, 21]. This trend continues to be facilitated by the COVID-19 pandemic, which has prompted digitization of government communications and transactions at an unprecedented rate and urged many police agencies to shift their community engagement activities online through social media platforms [19].

As a direct communication channel, social media allows the police to bypass traditional news media and reach a wider audience at a low cost and with greater efficiency [22, 23]. Policing scholarship has established that law enforcement agencies commonly seek to gather intelligence, enhance crime prevention and investigation, humanize the agency, engage in image-building activities, and improve their relations with the public through social media usage, which are consistent with the overall goals of community-oriented policing [11, 14, 24, 25]. At times of immediate crisis, police social media usage has the advantages over traditional news outlets to deliver instant messages to the mass. By exerting authority and providing immediate responses under exceptional circumstances, police agencies’ social media accounts often become the trusted source for information and can garner wide societal attention. Such instances were found during natural disasters, demonstration and social riots, terrorist attacks, among others [8, 26–28].

Nonetheless, the actual impact of social media usage in transforming police work and remediating police-community relations well depends on the way in which police agencies use it. Prior research suggests a variability across law enforcement agencies in social media usage, depending on agency organizational goals and pre-existing communication strategies [9]. This may be particularly evident in crisis situations. The crisis communication literature suggests that image-making and repair are one main motivation behind individual or organizational responses to crises [29]. Image is considered threatened when an organization or individual has committed or was responsible for an offensive act. Specifically, image repair theory identifies several approaches in response to accusations or damages including denial, evasion of responsibility (e.g., provocation, defeasibility, accident, or good intentions), reducing offensiveness of event (e.g., bolstering, minimization, differentiation, etc.), and mortification and corrective action attempt to repair an image without directly dealing with blame or offensiveness [30].

While social media may be used to promote a more open culture in police departments [31], social media platforms such as Twitter may also be used to publicize police-curated content unfiltered by traditional mass media, serving the purposes of deflecting institutional change (e.g., through socialization and/or legitimation) and mediating public pressure for reform. In a case study examining the New York Police Department (NYPD)’s daily Twitter posts in 2018 and an in-depth analysis of public reactions on Twitter to a contested NYPD shooting (i.e., the killing of Saheed Vassell), Cheng concluded that police social media usage represents “selective transparency” and mainly provides police with the technological capacity to “shape social memories while avoiding various forms of public accountability” (p.413) [13]. In the case of George Floyd, police as a profession have received heavy criticism for the long-standing racial disparities in policing outcomes and a string of fatal encounters between police and black citizens in recent years (e.g., Michael Brown, Freddie Gray, Breonna Taylor, etc.). As such, most U.S. police agencies would feel compelled to respond to the killing of George Floyd and associated protest activities through an image repair angle (e.g., emphasizing their role in fighting crime to ease public outrage or devoting an increased share of social media posts to racial justice related posts as a corrective action).

### Current study

The bulk of research on police use of social media has emerged within the past decade, scattered in such areas as criminology, sociology, public administration, communication, and information technology. It is far from clear what constitutes responsible and effective police public engagement on social media and whether actual uses of social media by police live up to the ideal of a community-oriented policing approach or mainly serve self-interested purposes [14, 16, 32, 33]. This body of research stays highly exploratory and is conducted typically on a small sample of agencies that limits their generalizability [7, 24, 33–35]. Narrowing this research gap has important implications to the study of innovative, sustainable ways through which police improve their engagement with targeted groups and the broader audiences. Expanding research on police use of social media also propels understanding of the utility (or lack thereof) of social media as a communication strategy for public or government agencies like law enforcement.

The current study seeks to examine how law enforcement agencies across the nation reacted on social media following a major legitimacy crisis. This inquiry is further situated within the context of a global pandemic that has pushed digital communication to the forefront. Specifically, we identified 115 large police departments in the U.S. with a regular presence on Twitter. We collected their tweets over a 4-month period between 4/1/2020 and 7/31/2020, covering critical periods before and after the killing of George Floyd and the nationwide protests that followed. With this, we aim to understand whether and how police agencies (both individually and as an aggregate) leveraged social media to respond to the social protests directed at the police. By combining a host of items that capture police activities on social media and public reactions to their activities, we created a single index to indicate how well police agencies engaged (or governed in a more neutral sense) the public on Twitter during the George Floyd protests.

## Materials and methods

### Data and sample

Using the 2016 Law Enforcement Management and Administrative Statistics (LEMAS) survey [36], we identified 139 large law enforcement agencies in the U.S. with more than 300 sworn officers and serving a population of 300,000 people and more. We focus on large agencies because they are most likely to have a regular social media presence, thus providing sufficient social media posts for analysis. The screening criteria were used in efforts to attain a meaningful and manageable sample of large U.S. police departments. We located Twitter handles of 137 (out of 139) law enforcement agencies. For police agencies with multiple official Twitter accounts, we selected only the main account with the greatest number of followers (also tweets and replies). All tweets from these agencies were fetched through Twitter’s Application Programming Interface (API) using R package *rtweet* on August 25th, 2020 [37]. Twitter handles of each of the 137 law enforcement agencies were used in the *get_timeline* function. Our data collection method complied with Twitter’s terms and conditions. We set the study period from April 1st, 2020 to July 31st, 2020 (approximately two months before and after the killing of George Floyd). This four-month study period allowed us enough data to analyze police Twitter usage before and after the killing of George Floyd and lessened the influence of the onset of the COVID-19 pandemic on police presence on social media. We excluded law enforcement agencies posting fewer than 50 tweets (including replies but excluding retweets) during this period. The final analysis sample included 115 law enforcement agencies and 38,701 tweets over a 4-month period. A complete list of these law enforcement agencies is included in S1 Table.

It is worth noting that we only examined police use of Twitter in the study due to data availability constraints. Twitter reaches between one-fifths and one-quarter of the U.S. population, and its users are younger, more likely to identify as Democrats, more highly educated and have higher incomes than U.S. adults overall [38]. Thus, the findings should be interpreted with caution due to the non-coverage of other social media platforms used by law enforcement agencies (e.g., Facebook or Nextdoor) and the demographics of Twitter users.

### Data analysis

Data analysis proceeded in three main steps. First, descriptive patterns were presented to show the frequency, public reactions (i.e., favorite and retweet counts), and emotions (characterized by pre-existing sentiment lexicon and metric) expressed in the tweets of the 115 large U.S. police departments before and after the killing of George Floyd. Second, a supervised machine learning algorithm was trained to categorize each of these tweets according to a 7-category scheme. The 7 categories are: 1) civil unrest related; 2) COVID-19 related; 3) police gathering of information; 4) police communication of administrative and mundane information; 5) police communication of traffic information; 6) police communication of case updates; and 7) community engagement and outreach. The categorization scheme was constructed based on previous studies of police social media usage, consultation of leading policing scholars and practitioners, and the focus of the current study [6, 22, 39]. Detailed categorization and exemplary tweets can be seen in S2 Table. Changes in the categories or focal issues of police departments tweets before and after the killing were analyzed. Specifically, to train the multiclass classifier, a random subset of 5,000 tweets were sampled and manually labeled into one of the 7 pre-defined categories. Each author independently labeled these tweets and the intercoder reliability was about 0.70. Discrepancies were identified, discussed, and resolved (i.e., agreement on the final categorizations). The random forest classifier was evaluated with the labeled tweets (with a 75/25 split) and then applied to the entire set of tweets for classification. Additional technical details can be found in S3 Table. Third, to assess adjustments of Twitter usage made by each of the 115 law enforcement agencies before and after the killing, we analyzed and ranked changes in the frequency, public reactions, and proportions of different categories of tweets. In addition, rankings for individual items were averaged to derive an overall ranking gauging police agencies’ performance on Twitter following the George Floyd protests. Data analysis was performed using R, version 4.0.2 in 2021.

## Results

### Descriptive analysis of aggregate police tweets

**Fig 1:**
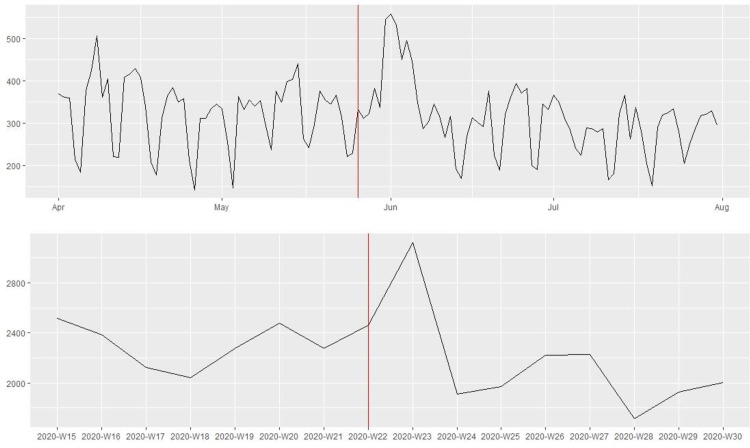
The number of tweets posted by the 115 major police departments in the U.S. before and after the killing of George Floyd on May 25^th^, 2020 (above: Daily information; bottom: Weekly information).

**Fig 2:**
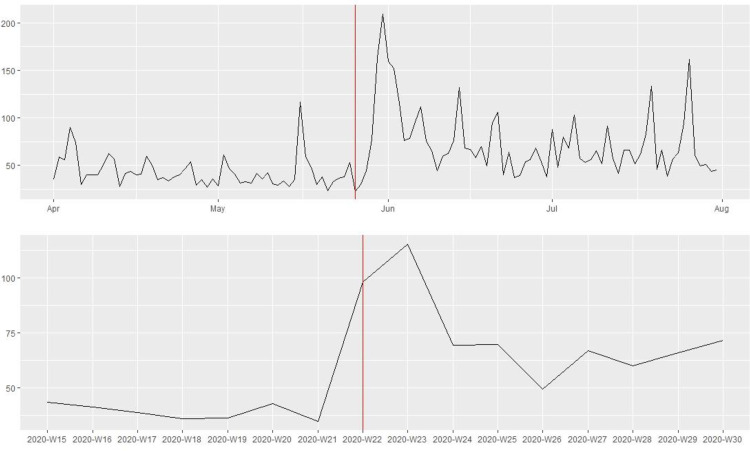
Public reactions (i.e., average number of favorites per tweet) to tweets posted by the 115 major police departments in the U.S. before and after the killing of George Floyd on May 25^th^, 2020 (above: Daily information; bottom: Weekly information).

**Fig 3:**
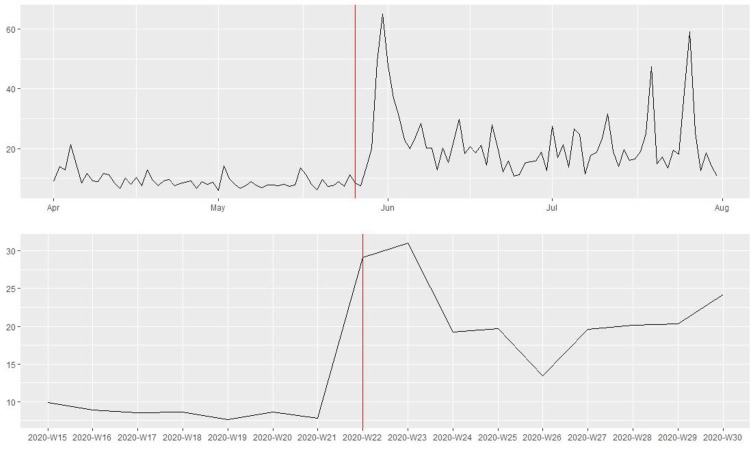
Public reactions (i.e., average number of retweets per tweet) to tweets posted by the 115 major police departments in the U.S. before and after the killing of George Floyd on May 25^th^, 2020 (above: Daily information; bottom: Weekly information).

Fig 1 shows that the 115 law enforcement agencies in our sample posted substantially more tweets in the week following the killing of George Floyd on May 25th, 2020. Yet, the number of tweets dropped to the pre-killing level after one week. Figs 2 and 3 present the average number of favorites and retweets received per tweet by the 115 law enforcement agencies before and after the killing. There was a noticeable increase in citizen reactions to police tweets immediately after the killing, and this trend lasted at least until the end of July 2020. To reduce the influence of “outliers”, tweets that received a favorite or retweet count exceeding three standard deviations above the mean were excluded. In the robustness check, substantively similar patterns were observed without excluding the outliers.

**Fig 4:**
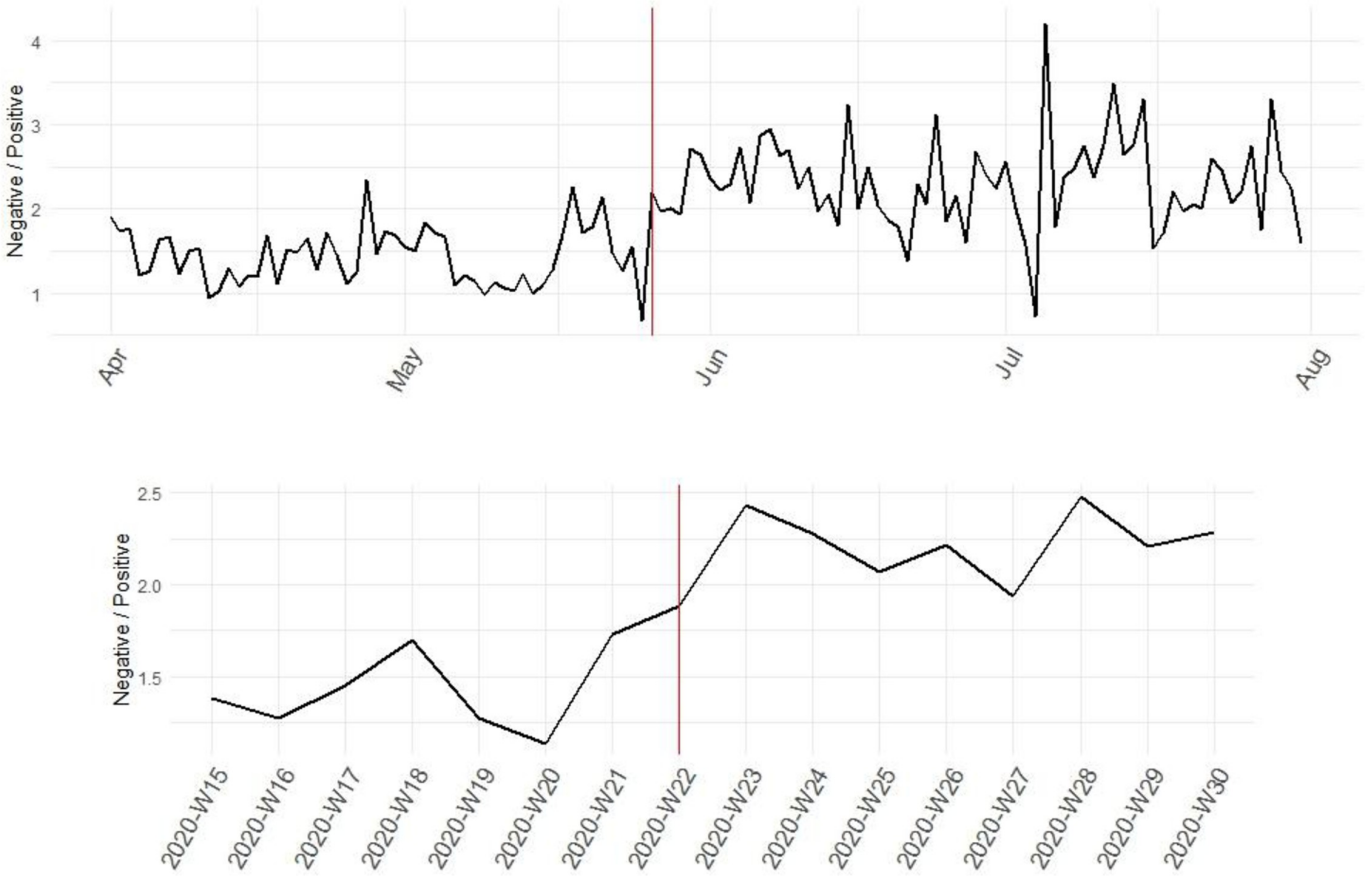
The negative-to-positive words ratio in the tweets of the 115 major police departments in the U.S. before and after the killing of George Floyd on May 25^th^, 2020 (above: Daily information; bottom: Weekly information).

**Fig 5:**
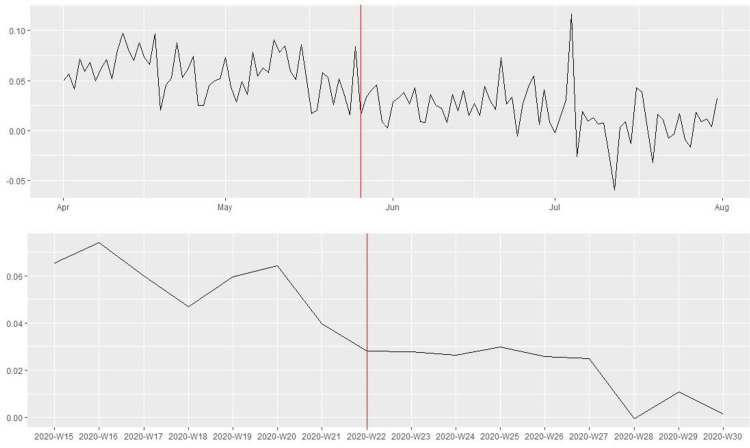
Emotional valence in the tweets of the 115 major police departments in the U.S. before and after the killing of George Floyd on May 25^th^, 2020 (above: Daily information; bottom: Weekly information).

Using the Bing sentiment lexicon—a widely used general purpose English lexicon that detects the sentiment of words through a dictionary lookup and classifies words as being “positive” or “negative” [40], Fig 4 shows that police-generated tweets during the study period were more likely to include words indicating a negative emotion than words expressing a positive emotion. The negative-to-positive words ratio further increased following the killing of George Floyd. Fig 5 depicts *sentence-level* emotional valence (i.e., the value associated with a stimulus as expressed on a continuum from pleasant to unpleasant or from attractive to aversive) in the tweets using Rinker’s *sentimentr* package. The package balances accuracy (e.g., considering valence shifters) and speed in calculating text polarity sentiment in the English language at the sentence level [41]. Consistent with Fig 4, there was a decrease in the “pleasantness” or “attractiveness” expressed in the tweets over the study period. Exemplary tweets illustrating sentence-level pleasant or attractive versus unpleasant or aversive emotion can be seen in S4 Table.

### Focal issues of police tweets

Table 1 reports the accuracy and kappa of the multiclass random forest classifier. Ten-fold cross-validation indicates an overall accuracy of 0.814 and a kappa value of 0.767. When applying the classifier to the split test set, the accuracy was 0.808 and the kappa value equaled to 0.758, indicating a reasonably high accuracy. By-class accuracy was also acceptable for all sub-categories in cross-validation and when applying to the split test set. Supplementary details about the results of the multiclass random forest classifier can be found in S5 Table. With the trained classifier, 37,899 tweets were classified into the 7 pre-defined categories. The number of tweets reduced from 38,701 to 37,899 because text pre-processing removed tweets that only contained hyperlinks, digits/numbers, images, videos, or stop words. Table 2 shows the number of tweets by categories. Consistent with prior research, the most frequent categories were for community engagement and outreach purpose and for case updates.

**Table 1:** Accuracy and kappa of the random forest classifier.

	Kappa	Accuracy (overall)	Accuracy (c1)	Accuracy (c2)	Accuracy (c3)	Accuracy (c4)	Accuracy (c5)	Accuracy (c6)	Accuracy (c7)
Cross validation	0.767	0.814	0.782	0.739	0.819	0.646	0.794	0.811	0.907
Split test set	0.758	0.808	0.766	0.739	0.866	0.580	0.759	0.819	0.901

*Note*: The 7 categories (or focal issues) are: 1) civil unrest related; 2) COVID-19 related; 3) police gathering of information; 4) police communication of administrative and mundane information; 5) police communication of traffic information; 6) police communication of case updates; and 7) community engagement and outreach.

**Table 2:** Number of tweets classified into each category.

	Frequency (overall)	Percentage (overall)	Frequency (pre-kill)	Percentage (pre-kill)	Frequency (post-kill)	Percentage (post-kill)
Class 1	2207	5.82%	45	0.26%	2162	10.58%
Class 2	2466	6.51%	1993	11.42%	473	2.31%
Class 3	4551	12.01%	1800	10.31%	2751	13.46%
Class 4	4459	11.77%	2082	11.93%	2377	11.63%
Class 5	3054	8.06%	1263	7.23%	1791	8.76%
Class 6	7602	20.06%	3046	17.45%	4556	22.29%
Class 7	13560	35.78%	7228	41.40%	6332	30.98%
	37899	100%	17457	100%	20442	100%

*Note*: The 7 categories (or focal issues) are: 1) civil unrest related; 2) COVID-19 related; 3) police gathering of information; 4) police communication of administrative and mundane information; 5) police communication of traffic information; 6) police communication of case updates; and 7) community engagement and outreach.

**Fig 6:**
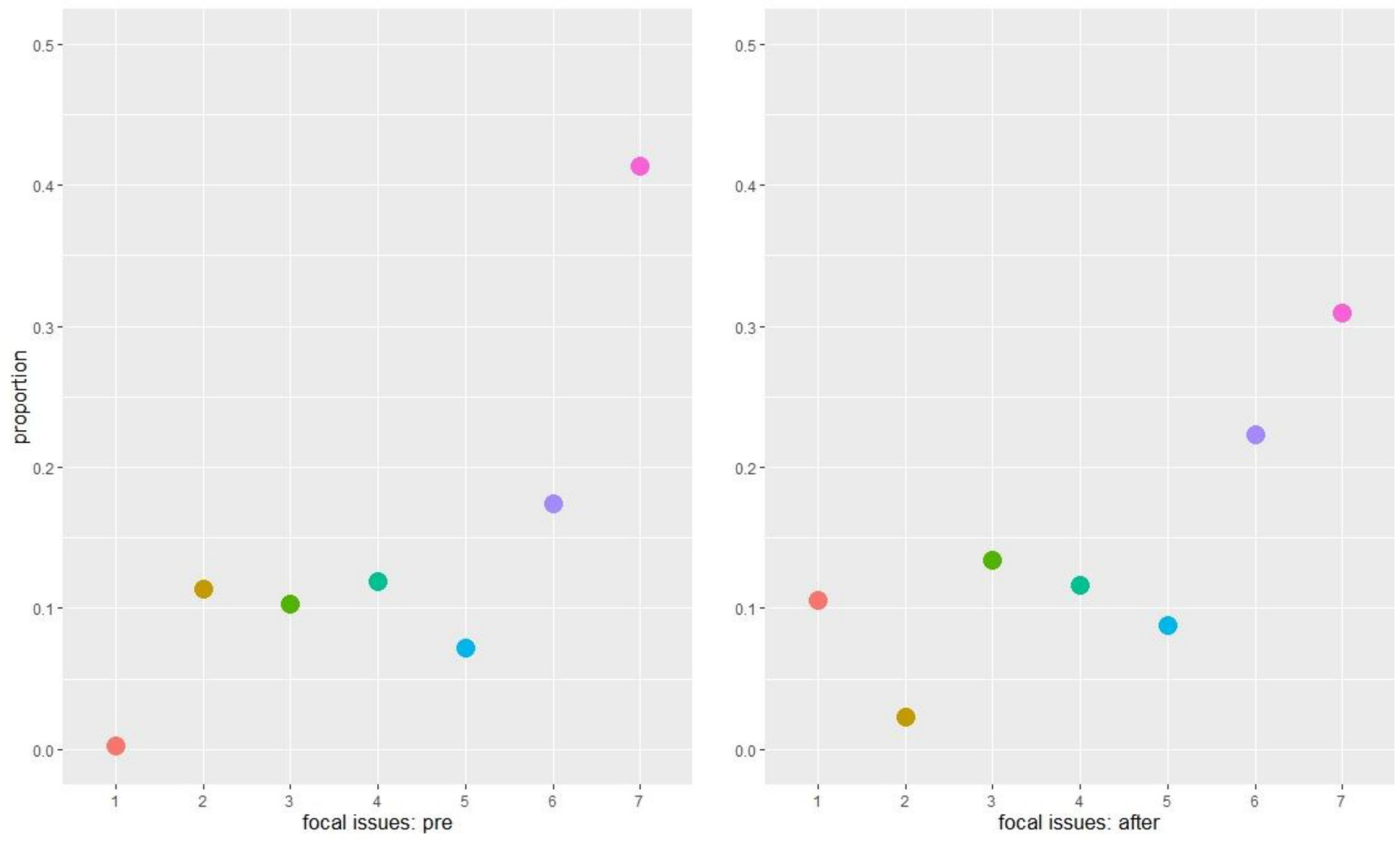
Proportions of focal issues mentioned in the tweets of the 115 major police departments in the U.S. before and after the killing of George Floyd on May 25^th^, 2020. Focal issues: (1) civil unrest related; (2) COVID-19 related; (3) police gathering of information; (4) police communication of administrative and mundane information; (5) police communication of traffic information; (6) police communication of case updates; and (7) community engagement and outreach.

**Fig 7:**
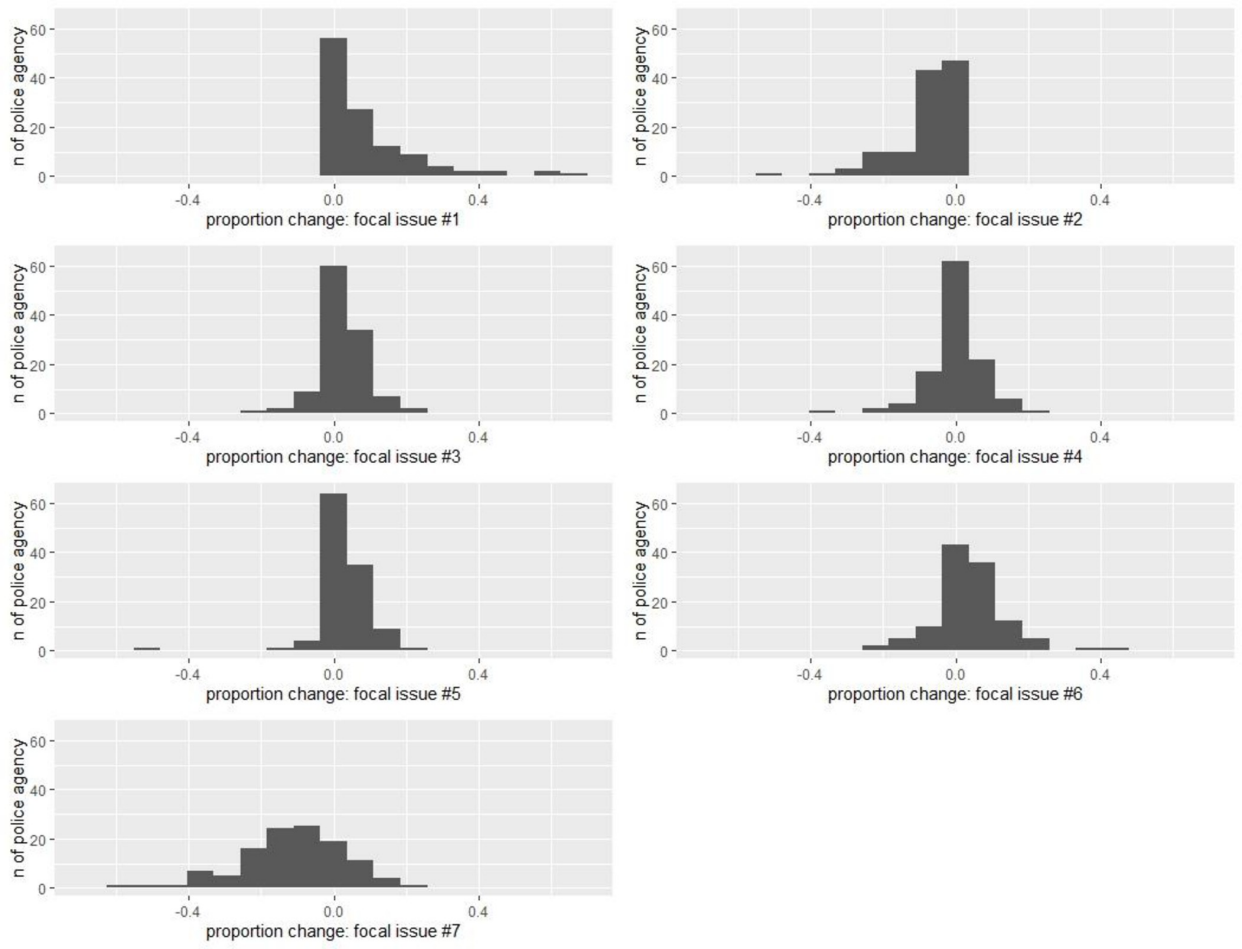
Distribution of police departments in relation to the changes in the focal issues of tweets posted by the 115 major police departments in the U.S. after the killing of George Floyd on May 25^th^, 2020. Focal issues: (1) civil unrest related; (2) COVID-19 related; (3) police gathering of information; (4) police communication of administrative and mundane information; (5) police communication of traffic information; (6) police communication of case updates; and (7) community engagement and outreach.

**Fig 8:**
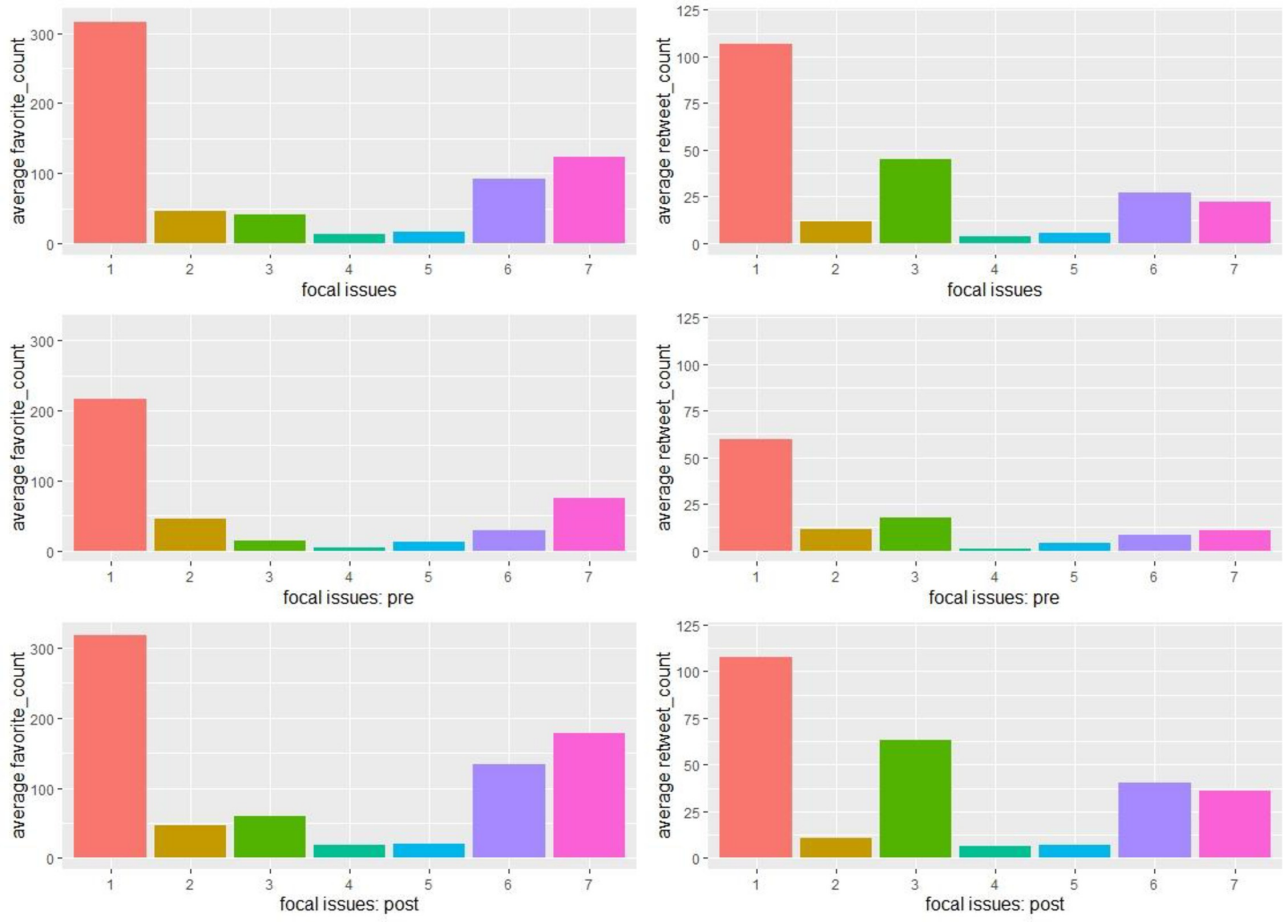
Public reactions by focal issues of the tweets posted by the 115 major police departments in the U.S. before and after the killing of George Floyd on May 25^th^, 2020. Focal issues: (1) civil unrest related; (2) COVID-19 related; (3) police gathering of information; (4) police communication of administrative and mundane information; (5) police communication of traffic information; (6) police communication of case updates; and (7) community engagement and outreach.

Fig 6 displays the proportions of focal issues mentioned in the tweets before and after the killing of George Floyd. There was a significant increase in the proportion of tweets related to civil unrest in the post-killing period and a significant decrease in the proportion of tweets that were COVID-19 related. More tweets were posted about case updates in the post-killing period, whereas fewer tweets were posted for community engagement and outreach purpose. Fig 7 further displays the distribution of police departments in relation to the changes in the focal issues of tweets. For instance, most police departments increased their posting of civil unrest related tweets (focal issue #1) and decreased the posting of COVID-19 related tweets (focal issue #2) in the post-killing period, whereas the distribution is more bell-shaped when looking at changes in tweets of community engagement and outreach (focal issue #7). Fig 8 shows public reactions by focal issues before and after the killing. The left panel shows that tweets related to civil unrest and community engagement and outreach received the highest average number of favorites per tweet. The pattern became more evident in the post-killing period. The right panel illustrates that tweets related to civil unrest and police gathering of information received the highest average number of retweets per tweet. Again, police audiences on Twitter were more likely to disseminate such information in the post-killing period.

### Adjustments of individual police departments Twitter usage

Tables 3–12 illustrate how each of the 115 law enforcement agencies adjusted their Twitter usage before and after the killing of George Floyd. To adjust for baseline levels of tweeting practices across police departments, the 115 law enforcement agencies were divided into two groups. Across the 115 law enforcement agencies, the mean was 155 and the median was 114 tweets in the pre-killing period. We made the cut-point at 110 tweets in the pre-killing period to create the two groups. The first group included the more active agencies, namely, the 60 agencies which posted, on average, at least 2 tweets per day during the pre-killing period (i.e., the higher-use group). The second group included the other 55 agencies which were less active on Twitter during the pre-killing period (i.e., the lower-use group). By dividing the agencies into the higher-use and lower-use groups, we balanced the raw and percentage changes when ranking the agencies and partially adjusted for potential influences of agency/personnel size and jurisdiction population (i.e., agency-level factors) on police social media usage.

**Table 3:** Agencies (in the higher-use group) ranked by the increase in the number of tweets posted before and after the killing.

#	Agency name	Total number of tweets pre-killing	Total number of tweets post-killing	Number of tweets per day pre-killing	Number of tweets per day post-killing	Frequency change	Percentage change
1	Aurora Police Dept.	152	477	2.76	7.12	4.36	158
2	Portland Police Dept.	305	915	5.55	13.7	8.11	146
3	Milwaukee Police Dept.	428	812	7.78	12.1	4.34	55.7
4	Seattle Police Dept.	121	227	2.2	3.39	1.19	54
5	Charlotte-Mecklenburg Police Dept.	196	325	3.56	4.85	1.29	36.1
6	Memphis Police Dept.	118	191	2.15	2.85	0.705	32.9
7	Jefferson County(CO) Sheriff’s Office	131	208	2.38	3.1	0.723	30.3
8	Omaha Police Dept.	216	341	3.93	5.09	1.16	29.6
9	Kansas City Police Dept.	275	426	5	6.36	1.36	27.2
10	Montgomery County(MD) Police Dept.	181	272	3.29	4.06	0.769	23.4
11	Denver Police Dept.	847	1259	15.4	18.8	3.39	22
12	Austin Police Dept.	168	243	3.05	3.63	0.572	18.7
13	Baltimore Police Dept.	163	232	2.96	3.46	0.499	16.8
14	D.C. Metropolitan Police Dept.	778	1089	14.1	16.3	2.11	14.9
15	Raleigh Police Dept.	172	227	3.13	3.39	0.261	8.34
16	Prince William County Police Dept.	257	338	4.67	5.04	0.372	7.96
17	Gwinnett County Police Dept.	120	157	2.18	2.34	0.161	7.4
18	Oklahoma City Police Dept.	182	224	3.31	3.34	0.0342	1.03
19	Washington County Sheriff’s Office	129	154	2.35	2.3	-0.0469	-2
20	Bakersfield Police Dept.	135	161	2.45	2.4	-0.0516	-2.1
21	Palm Beach County Sheriff’s Office	180	212	3.27	3.16	-0.109	-3.32
22	Dallas Police Dept.	425	493	7.73	7.36	-0.369	-4.78
23	Jacksonville Sheriff’s Office	143	164	2.6	2.45	-0.152	-5.86
24	Jefferson County(AL) Sheriff’s Dept.	126	144	2.29	2.15	-0.142	-6.18
25	Chicago Police Dept.	168	191	3.05	2.85	-0.204	-6.67
26	Hillsborough County Sheriff’s Office	233	264	4.24	3.94	-0.296	-6.99
27	Orange County(FL) Sheriff’s Office	576	638	10.5	9.52	-0.95	-9.07
28	Manatee County(FL) Sheriff’s Office	116	128	2.11	1.91	-0.199	-9.42
29	Honolulu Police Dept.	196	215	3.56	3.21	-0.355	-9.95
30	Houston Police Dept.	453	485	8.24	7.24	-0.998	-12.1
31	Loudoun County Sheriff’s Office	114	121	2.07	1.81	-0.267	-12.9
32	Bernalillo County Sheriff’s Dept.	210	221	3.82	3.3	-0.52	-13.6
33	Columbus Police Dept.	296	311	5.38	4.64	-0.74	-13.8
34	Boston Police Dept.	214	224	3.89	3.34	-0.548	-14.1
35	Prince George’s County Police Dept.	220	228	4	3.4	-0.597	-14.9
36	Douglas County(CO) Sheriff’s Office	118	115	2.15	1.72	-0.429	-20
37	Broward County Sheriff’s Office	173	168	3.15	2.51	-0.638	-20.3
38	San Diego County Sheriff’s Dept.	139	133	2.53	1.99	-0.542	-21.5
39	Phoenix Police Dept.	184	173	3.35	2.58	-0.763	-22.8
40	Alameda County Sheriff’s Office	181	168	3.29	2.51	-0.783	-23.8
41	El Paso County Sheriff’s Office	133	121	2.42	1.81	-0.612	-25.3
42	Fairfax County Police Dept.	367	318	6.67	4.75	-1.93	-28.9
43	San Diego Police Dept.	226	195	4.11	2.91	-1.2	-29.2
44	Harris county Sheriff’s Office	224	192	4.07	2.87	-1.21	-29.6
45	Tampa Police Dept.	110	94	2	1.4	-0.597	-29.9
46	Montgomery County (TX) Sheriff’s Office	125	104	2.27	1.55	-0.72	-31.7
47	Fort Worth Police Dept.	197	160	3.58	2.39	-1.19	-33.3
48	Las Vegas Police Dept.	112	90	2.04	1.34	-0.693	-34
49	Los Angeles County Sheriff’s Dept.	266	212	4.84	3.16	-1.67	-34.6
50	Henrico County Police Dept.	145	114	2.64	1.7	-0.935	-35.5
51	Sacramento Police Dept.	153	114	2.78	1.7	-1.08	-38.8
52	Anne Arundel Police Dept.	198	144	3.6	2.15	-1.45	-40.3
53	Baltimore County Police Dept.	220	160	4	2.39	-1.61	-40.3
54	New York Police Dept.	663	432	12.1	6.45	-5.61	-46.5
55	Richland County Sheriff’s Dept.	687	443	12.5	6.61	-5.88	-47.1
56	Chesterfield County Police Dept.	119	67	2.16	1	-1.16	-53.8
57	Miami Police Dept.	495	270	9	4.03	-4.97	-55.2
58	Pierce County Sheriff’s Dept.	153	78	2.78	1.16	-1.62	-58.2
59	Wichita Police Dept.	166	81	3.02	1.21	-1.81	-59.9
60	Pinellas County Police Dept.	228	41	4.15	0.612	-3.53	-85.2

**Table 4:** Agencies (in the lower-use group) ranked by the increase in the number of tweets posted before and after the killing.

#	Agency name	Total number of tweets pre-killing	Total number of tweets post-killing	Number of tweets per day pre-killing	Number of tweets per day post-killing	Frequency change	Percentage change
1	Long Beach Police Dept.	41	233	0.745	3.48	2.73	367
2	Cleveland Police Dept.	18	79	0.327	1.18	0.852	260
3	Saint Paul Police Dept.	36	109	0.655	1.63	0.972	149
4	Pima County Sheriff’s Dept.	66	173	1.2	2.58	1.38	115
5	Minneapolis Police Dept.	19	48	0.345	0.716	0.371	107
6	Virginia Beach Police Dept.	43	106	0.782	1.58	0.8	102
7	Atlanta Police Dept.	33	71	0.6	1.06	0.46	76.6
8	Louisville Metropolitan Police Dept.	21	45	0.382	0.672	0.29	75.9
9	Philadelphia Police Dept.	62	130	1.13	1.94	0.813	72.1
10	Tulsa Police Dept.	50	104	0.909	1.55	0.643	70.7
11	Los Angeles Police Dept.	91	181	1.65	2.7	1.05	63.3
12	Lexington Police Dept.	38	73	0.691	1.09	0.399	57.7
13	San Antonio Police Dept.	21	38	0.382	0.567	0.185	48.5
14	Riverside(CA) Police Dept.	31	56	0.564	0.836	0.272	48.3
15	Arlington Police Dept.	88	155	1.6	2.31	0.713	44.6
16	Washoe County Sheriff’s Office	81	140	1.47	2.09	0.617	41.9
17	Detroit Police Dept.	43	74	0.782	1.1	0.323	41.3
18	Shelby County(TN) Sheriff’s Office	45	76	0.818	1.13	0.316	38.6
19	San Jose Police Dept.	21	35	0.382	0.522	0.141	36.8
20	Pittsburgh Bureau of Police	80	128	1.45	1.91	0.456	31.3
21	Travis County Sheriff’s Office	20	32	0.364	0.478	0.114	31.3
22	Collier County Sheriff’s Office	50	78	0.909	1.16	0.255	28.1
23	Orange County(CA) Sheriff’s Dept.	58	88	1.05	1.31	0.259	24.5
24	Ventura County Sheriff’s Office	78	117	1.42	1.75	0.328	23.1
25	Stockton(CA) Police Dept.	36	53	0.655	0.791	0.136	20.9
26	Oakland(CA) Police Dept.	67	95	1.22	1.42	0.2	16.4
27	Colorado Springs Police Dept.	85	118	1.55	1.76	0.216	14
28	Adams County Sheriff’s Office	97	134	1.76	2	0.236	13.4
29	El Paso Police Dept.	65	81	1.18	1.21	0.0271	2.3
30	Santa Ana Police Dept.	99	122	1.8	1.82	0.0209	1.16
31	Metropolitan Nashville Police Dept.	105	118	1.91	1.76	-0.148	-7.75
32	San Francisco Police Dept.	92	101	1.67	1.51	-0.165	-9.88
33	Arapahoe County Sheriff’s Office	68	73	1.24	1.09	-0.147	-11.9
34	St. Louis Police Dept.	84	90	1.53	1.34	-0.184	-12
35	St. Louis County Police Dept.	93	99	1.69	1.48	-0.213	-12.6
36	Dekalb County Police Dept.	76	74	1.38	1.1	-0.277	-20.1
37	Osceola County Sheriff’s Office	70	67	1.27	1	-0.273	-21.4
38	Franklin County Sheriff’s Office	75	71	1.36	1.06	-0.304	-22.3
39	Corpus Christi Police Dept.	45	42	0.818	0.627	-0.191	-23.4
40	Tucson Police Dept.	43	37	0.782	0.552	-0.23	-29.4
41	Kern County Sheriff’s Dept.	91	75	1.65	1.12	-0.535	-32.3
42	Lee County(FL) Sheriff’s Office	81	66	1.47	0.985	-0.488	-33.1
43	Anaheim Police Dept.	62	50	1.13	0.746	-0.381	-33.8
44	New Castle County Police Dept.	58	46	1.05	0.687	-0.368	-34.9
45	Volusia County Sheriff’s Office	95	71	1.73	1.06	-0.668	-38.6
46	Seminole County Sheriff’s Office	33	23	0.6	0.343	-0.257	-42.8
47	Hennepin County Sheriff’s Office	61	40	1.11	0.597	-0.512	-46.2
48	Suffolk County Police Dept.	80	51	1.45	0.761	-0.693	-47.7
49	Albuquerque Police Dept.	79	49	1.44	0.731	-0.705	-49.1
50	Howard County Police Dept.	50	30	0.909	0.448	-0.461	-50.7
51	Santa Clara County Sheriff’s Office	32	19	0.582	0.284	-0.298	-51.3
52	Sacramento County Sheriff’s Office	37	21	0.673	0.313	-0.359	-53.4
53	Unified (Salt Lake, Utah) Police Dept.	35	18	0.636	0.269	-0.368	-57.8
54	Mesa Police Dept.	107	47	1.95	0.701	-1.24	-63.9
55	East Baton Rouge Sheriff’s Office	62	24	1.13	0.358	-0.769	-68.2

**Table 5:** Agencies (in the higher-use group) ranked by the increase in the received favorites per tweet before and after the killing.

#	Agency name	Average number of favorites received per tweet pre-killing	Average number of favorites received per tweet post-killing	Raw change	Percentage change
1	Portland Police Dept.	35.8	236	200	559
2	Seattle Police Dept.	65.3	380	314	482
3	Charlotte-Mecklenburg Police Dept.	9.77	47.1	37.4	383
4	Tampa Police Dept.	22.9	92.4	69.5	304
5	San Diego Police Dept.	55.2	197	142	257
6	Austin Police Dept.	21.2	75.2	54.1	255
7	Pinellas County Police Dept.	6.77	21	14.2	209
8	Milwaukee Police Dept.	5.64	17.4	11.8	209
9	Columbus Police Dept.	22.6	61.4	38.8	172
10	Wichita Police Dept.	16.5	44.7	28.3	172
11	Dallas Police Dept.	28.6	73.5	44.9	157
12	Oklahoma City Police Dept.	26	65.7	39.7	153
13	Alameda County Sheriff’s Office	26.3	59.6	33.3	127
14	New York Police Dept.	103	231	128	124
15	Omaha Police Dept.	33.1	70.6	37.4	113
16	D.C. Metropolitan Police Dept.	14.9	31.2	16.3	109
17	Miami Police Dept.	14.4	30.1	15.6	108
18	Denver Police Dept.	7.88	16.4	8.47	108
19	Hillsborough County Sheriff’s Office	21.9	42.7	20.8	94.7
20	Baltimore Police Dept.	21	40.5	19.5	93.2
21	Gwinnett County Police Dept.	18	32.5	14.4	79.9
22	Boston Police Dept.	78	137	59.4	76.1
23	Sacramento Police Dept.	29.8	52.4	22.6	75.9
24	Phoenix Police Dept.	84.5	148	63.9	75.6
25	Memphis Police Dept.	3.53	6.11	2.58	73.3
26	Houston Police Dept.	48.6	82.8	34.2	70.5
27	Fort Worth Police Dept.	54.3	90.7	36.4	67
28	Jefferson County(AL) Sheriff’s Dept.	38	63.4	25.3	66.7
29	Chicago Police Dept.	112	176	63.5	56.6
30	Chesterfield County Police Dept.	10.8	16.5	5.75	53.4
31	Aurora Police Dept.	19.2	28.1	8.93	46.5
32	San Diego County Sheriff’s Dept.	36.3	51.6	15.3	42.2
33	Douglas County(CO) Sheriff’s Office	33.2	46.5	13.3	40.1
34	Kansas City Police Dept.	96.3	134	37.9	39.3
35	Las Vegas Police Dept.	107	145	38.4	35.9
36	Prince George’s County Police Dept.	13.6	18.2	4.65	34.2
37	Fairfax County Police Dept.	27.3	34.8	7.42	27.2
38	Pierce County Sheriff’s Dept.	82.4	103	20.9	25.3
39	Richland County Sheriff’s Dept.	154	186	32.5	21.2
40	Los Angeles County Sheriff’s Dept.	57.1	68.1	11	19.2
41	Prince William County Police Dept.	12.8	14.9	2.07	16.2
42	Harris county Sheriff’s Office	33.2	38.2	4.98	15
43	Honolulu Police Dept.	11	12.6	1.61	14.6
44	Palm Beach County Sheriff’s Office	64.7	73.7	8.97	13.9
45	Orange County(FL) Sheriff’s Office	22.3	24.6	2.34	10.5
46	Baltimore County Police Dept.	12.8	14	1.16	9.01
47	Bakersfield Police Dept.	7.65	8.17	0.522	6.82
48	Montgomery County (TX) Sheriff’s Office	5.10	5.43	0.337	6.61
49	Jefferson County(CO) Sheriff’s Office	42.3	44.8	2.49	5.87
50	Jacksonville Sheriff’s Office	43.2	45.4	2.22	5.14
51	Manatee County(FL) Sheriff’s Office	15.2	15.4	0.227	1.5
52	Loudoun County Sheriff’s Office	12.6	12.2	-0.354	-2.82
53	Raleigh Police Dept.	31.2	29.2	-2.02	-6.46
54	Montgomery County(MD) Police Dept.	22.4	20.9	-1.49	-6.63
55	Bernalillo County Sheriff’s Dept.	9.02	8.39	-0.635	-7.03
56	El Paso County Sheriff’s Office	29.6	23.7	-5.84	-19.7
57	Broward County Sheriff’s Office	78.3	60.8	-17.5	-22.4
58	Washington County Sheriff’s Office	53	40.6	-12.5	-23.5
59	Anne Arundel Police Dept.	22.1	15.7	-6.39	-28.9
60	Henrico County Police Dept.	8.12	5.51	-2.61	-32.1

**Table 6:** Agencies (in the lower-use group) ranked by the increase in the received favorites per tweet before and after the killing.

#	Agency name	Average number of favorites received per tweet pre-killing	Average number of favorites received per tweet post-killing	Raw change	Percentage change
1	Ventura County Sheriff’s Office	11.5	139	128	1117
2	Saint Paul Police Dept.	19	137	118	619
3	Atlanta Police Dept.	40	222	182	455
4	Minneapolis Police Dept.	68.5	347	279	407
5	Louisville Metropolitan Police Dept.	31.5	147	116	368
6	Unified (Salt Lake, Utah) Police Dept.	7.23	32.8	25.6	354
7	Philadelphia Police Dept.	23.7	102	78.6	331
8	Anaheim Police Dept.	40.4	164	124	306
9	Stockton(CA) Police Dept.	13.8	49.3	35.5	257
10	Lexington Police Dept.	15.6	55.6	40	256
11	St. Louis Police Dept.	34.6	111	76.3	221
12	Cleveland Police Dept.	56.6	172	116	204
13	Tulsa Police Dept.	82.6	237	155	187
14	Metropolitan Nashville Police Dept.	109	306	197	181
15	San Jose Police Dept.	57	138	81.3	143
16	Pima County Sheriff’s Dept.	10.3	24.7	14.4	139
17	Howard County Police Dept.	24	54.8	30.9	129
18	Albuquerque Police Dept.	59.9	136	76.4	127
19	Osceola County Sheriff’s Office	5.83	12.2	6.41	110
20	San Antonio Police Dept.	57.6	120	62.4	108
21	St. Louis County Police Dept.	58	120	61.8	107
22	Seminole County Sheriff’s Office	18.4	33	14.6	79.7
23	Oakland(CA) Police Dept.	42.1	72.6	30.5	72.4
24	Colorado Springs Police Dept.	32.6	50.9	18.3	56.1
25	Riverside(CA) Police Dept.	22.5	33.9	11.4	50.5
26	Los Angeles Police Dept.	308	447	139	45.1
27	Detroit Police Dept.	36.3	51.8	15.4	42.5
28	Santa Clara County Sheriff’s Office	37.3	53.1	15.8	42.4
29	Hennepin County Sheriff’s Office	18.9	26.5	7.57	40.1
30	Franklin County Sheriff’s Office	83.2	115	32.1	38.6
31	Long Beach Police Dept.	13.6	18.6	4.99	36.8
32	Santa Ana Police Dept.	27.9	38	10.1	36.1
33	San Francisco Police Dept.	53	68.5	15.6	29.4
34	Dekalb County Police Dept.	14.8	18.4	3.52	23.8
35	Mesa Police Dept.	48.7	59.8	11.1	22.9
36	Corpus Christi Police Dept.	18.3	22.2	3.9	21.3
37	Arapahoe County Sheriff’s Office	49.2	57.1	7.92	16.1
38	Sacramento County Sheriff’s Office	16.1	18.7	2.58	16
39	Arlington Police Dept.	50.3	55.3	4.97	9.88
40	Virginia Beach Police Dept.	17.8	19.3	1.5	8.44
41	Shelby County(TN) Sheriff’s Office	8.78	9.49	0.709	8.08
42	Suffolk County Police Dept.	41.4	43.1	1.68	4.05
43	Collier County Sheriff’s Office	15	15.3	0.268	1.78
44	Orange County(CA) Sheriff’s Dept.	93.5	93.4	-0.103	-0.11
45	Volusia County Sheriff’s Office	41.5	37.6	-3.88	-9.36
46	El Paso Police Dept.	170	152	-18.1	-10.6
47	Kern County Sheriff’s Dept.	5.09	4.47	-0.621	-12.2
48	Lee County(FL) Sheriff’s Office	21	18.3	-2.74	-13.1
49	Tucson Police Dept.	62.2	41.2	-21.1	-33.9
50	Washoe County Sheriff’s Office	29.8	18.7	-11.1	-37.3
51	Adams County Sheriff’s Office	35.4	20	-15.3	-43.3
52	Travis County Sheriff’s Office	49.6	26.6	-23.1	-46.4
53	Pittsburgh Bureau of Police	45.4	20.4	-25	-55.1
54	East Baton Rouge Sheriff’s Office	14.3	6.38	-7.93	-55.4
55	New Castle County Police Dept.	9.45	3.61	-5.84	-61.8

**Table 7:** Agencies (in the higher-use group) ranked by the increase in the received retweets per tweet before and after the killing.

#	Agency name	Average number of retweets received per tweet pre-killing	Average number of retweets received per tweet post-killing	Raw change	Percentage change
1	Portland Police Dept.	5.63	75.7	70	1245
2	Seattle Police Dept.	17	138	121	710
3	Wichita Police Dept.	4.69	19.1	14.4	306
4	Charlotte-Mecklenburg Police Dept.	3.89	15.4	11.5	295
5	San Diego Police Dept.	7.84	30.9	23	294
6	Tampa Police Dept.	4.94	19.1	14.2	288
7	Columbus Police Dept.	5.55	21.3	15.8	284
8	Sacramento Police Dept.	4.26	14.9	10.6	249
9	Alameda County Sheriff’s Office	6.86	22.9	16	234
10	New York Police Dept.	26.6	88.3	61.7	232
11	Austin Police Dept.	7.43	24.6	17.2	231
12	Baltimore Police Dept.	5.87	17.5	11.7	198
13	Miami Police Dept.	4.19	12.2	8.03	192
14	Dallas Police Dept.	6.79	19.7	12.9	191
15	Milwaukee Police Dept.	2.25	6.22	3.97	176
16	Aurora Police Dept.	4.64	12.5	7.88	170
17	Oklahoma City Police Dept.	7.41	19.8	12.4	167
18	Denver Police Dept.	1.71	4.27	2.55	149
19	Hillsborough County Sheriff’s Office	4.08	10.1	6.04	148
20	Phoenix Police Dept.	15.7	35.2	19.6	125
21	Chesterfield County Police Dept.	2.3	4.87	2.56	111
22	Las Vegas Police Dept.	24.2	48.3	24.1	99.7
23	Fort Worth Police Dept.	13.5	26.7	13.1	97.1
24	Omaha Police Dept.	2.9	5.48	2.58	89
25	Chicago Police Dept.	19.6	36.3	16.7	84.9
26	Fairfax County Police Dept.	5.37	9.36	3.99	74.2
27	D.C. Metropolitan Police Dept.	9.15	15.8	6.68	73
28	Douglas County(CO) Sheriff’s Office	4.99	8.62	3.63	72.6
29	Pinellas County Police Dept.	3.02	5.12	2.1	69.7
30	Bernalillo County Sheriff’s Dept.	1.34	2.22	0.879	65.7
31	Boston Police Dept.	15.2	25.2	9.96	65.5
32	Richland County Sheriff’s Dept.	11	17.8	6.83	62.3
33	Baltimore County Police Dept.	4.93	7.89	2.96	60.1
34	Palm Beach County Sheriff’s Office	16.6	25.6	8.96	54
35	Loudoun County Sheriff’s Office	2.31	3.5	1.2	51.9
36	Houston Police Dept.	15.6	23.3	7.64	48.8
37	Los Angeles County Sheriff’s Dept.	18.7	27.3	8.6	45.9
38	Gwinnett County Police Dept.	5.9	8.57	2.67	45.3
39	Memphis Police Dept.	3.86	5.27	1.42	36.7
40	Montgomery County (TX) Sheriff’s Office	1.38	1.83	0.443	32
41	Jacksonville Sheriff’s Office	18.8	24.5	5.7	30.3
42	Harris county Sheriff’s Office	10.1	13.1	3.03	30.1
43	Pierce County Sheriff’s Dept.	15.9	20.6	4.74	29.9
44	Jefferson County(CO) Sheriff’s Office	7.02	9.07	2.04	29.1
45	Montgomery County(MD) Police Dept.	10.8	14	3.12	28.8
46	Kansas City Police Dept.	18.3	23.5	5.23	28.7
47	Prince George’s County Police Dept.	11.7	14	2.31	19.7
48	Manatee County(FL) Sheriff’s Office	2.66	3.15	0.493	18.6
49	Prince William County Police Dept.	3.3	3.89	0.594	18
50	Jefferson County(AL) Sheriff’s Dept.	5.29	6.08	0.798	15.1
51	Orange County(FL) Sheriff’s Office	6.04	6.89	0.849	14.1
52	Washington County Sheriff’s Office	9.81	11.1	1.26	12.9
53	Raleigh Police Dept.	8.28	8.48	0.191	2.3
54	San Diego County Sheriff’s Dept.	16	16.3	0.286	1.79
55	El Paso County Sheriff’s Office	7.62	7.69	0.0619	0.812
56	Bakersfield Police Dept.	3.5	3.23	-0.266	-7.62
57	Broward County Sheriff’s Office	14.8	13.5	-1.33	-8.98
58	Henrico County Police Dept.	1.58	1.35	-0.228	-14.5
59	Honolulu Police Dept.	2.99	2.4	-0.59	-19.7
60	Anne Arundel Police Dept.	4.66	3.21	-1.45	-31.1

**Table 8:** Agencies (in the lower-use group) ranked by the increase in the received retweets per tweet before and after the killing.

#	Agency name	Average number of retweets received per tweet pre-killing	Average number of retweets received per tweet post-killing	Raw change	Percentage change
1	Ventura County Sheriff’s Office	2.81	43.4	40.6	1447
2	Louisville Metropolitan Police Dept.	7.62	66.3	58.6	770
3	Saint Paul Police Dept.	6	48.5	42.5	709
4	St. Louis Police Dept.	5.63	45	39.4	700
5	Sacramento County Sheriff’s Office	2.05	14	12	584
6	Anaheim Police Dept.	7.27	36.6	29.4	404
7	Unified (Salt Lake, Utah) Police Dept.	1.03	5.11	4.08	397
8	St. Louis County Police Dept.	6.05	28.5	22.5	371
9	Minneapolis Police Dept.	20.1	92.3	72.2	359
10	Albuquerque Police Dept.	9.33	42.2	32.9	353
11	Tulsa Police Dept.	14.8	65.4	50.6	343
12	Metropolitan Nashville Police Dept.	21.7	89.6	67.8	312
13	San Jose Police Dept.	8.14	31.2	23.1	284
14	Stockton(CA) Police Dept.	3.03	11.5	8.48	280
15	Philadelphia Police Dept.	14.4	52.3	37.9	263
16	Lexington Police Dept.	4.08	12.8	8.69	213
17	Detroit Police Dept.	6.6	18.5	11.9	180
18	Cleveland Police Dept.	31	84.1	53.1	171
19	Oakland(CA) Police Dept.	8.73	23.6	14.9	171
20	Colorado Springs Police Dept.	4.52	11.9	7.36	163
21	Pima County Sheriff’s Dept.	5.64	13.4	7.72	137
22	Dekalb County Police Dept.	7.11	15.8	8.65	122
23	Atlanta Police Dept.	37	80.1	43	116
24	Hennepin County Sheriff’s Office	3.46	7.48	4.02	116
25	Osceola County Sheriff’s Office	1.29	2.75	1.46	114
26	Mesa Police Dept.	10.1	21.6	11.5	114
27	Santa Clara County Sheriff’s Office	4.91	10.4	5.51	112
28	Howard County Police Dept.	6.24	12.9	6.69	107
29	San Antonio Police Dept.	17.6	35.3	17.7	100
30	Arapahoe County Sheriff’s Office	4.15	8.16	4.02	96.9
31	Seminole County Sheriff’s Office	3.7	6.96	3.26	88.2
32	Santa Ana Police Dept.	4.98	8.47	3.49	70
33	Virginia Beach Police Dept.	3.63	6.14	2.51	69.3
34	Los Angeles Police Dept.	86	137	50.8	59.1
35	San Francisco Police Dept.	11.6	17	5.39	46.3
36	Franklin County Sheriff’s Office	10.2	14.8	4.6	45.1
37	El Paso Police Dept.	35.8	51.2	15.4	43
38	Corpus Christi Police Dept.	6.73	9.14	2.41	35.8
39	Long Beach Police Dept.	3.9	5.07	1.17	30
40	Shelby County(TN) Sheriff’s Office	4.76	5.43	0.679	14.3
41	Pittsburgh Bureau of Police	8.6	9.57	0.97	11.3
42	Suffolk County Police Dept.	7.6	8.39	0.792	10.4
43	Volusia County Sheriff’s Office	8.91	9.69	0.785	8.81
44	Collier County Sheriff’s Office	3.84	4.1	0.263	6.84
45	Lee County(FL) Sheriff’s Office	3.07	3.03	-0.0438	-1.42
46	Kern County Sheriff’s Dept.	0.956	0.933	-0.0227	-2.38
47	Arlington Police Dept.	8.76	8.28	-0.477	-5.45
48	Washoe County Sheriff’s Office	4.88	4.41	-0.462	-9.48
49	Orange County(CA) Sheriff’s Dept.	16.4	14.6	-1.74	-10.6
50	Tucson Police Dept.	23.5	21	-2.56	-10.9
51	Adams County Sheriff’s Office	2.57	2.19	-0.38	-14.8
52	Riverside(CA) Police Dept.	10.8	8.79	-1.99	-18.5
53	East Baton Rouge Sheriff’s Office	2.44	1.75	-0.685	-28.1
54	New Castle County Police Dept.	2.76	1.26	-1.5	-54.3
55	Travis County Sheriff’s Office	10.8	4.38	-6.48	-59.7

**Table 9:** Agencies (in the higher-use group) ranked by the increase in posting category 1 (civil unrest related) tweets before and after the killing.

#	Agency name	Total number of tweets pre-killing	Total number of tweets post-killing	Number of C1 tweets pre-killing	Number of C1 tweets post-killing	Percentage of C1 tweets pre-killing	Percentage of C1 tweets post-killing	Percentage change
1	Portland Police Dept.	303	900	0	535	0	0.594	0.594
2	Dallas Police Dept.	425	491	0	193	0	0.393	0.393
3	Seattle Police Dept.	117	226	0	79	0	0.35	0.35
4	San Diego Police Dept.	200	191	0	57	0	0.298	0.298
5	Charlotte-Mecklenburg Police Dept.	195	314	2	89	0.0103	0.283	0.273
6	Raleigh Police Dept.	162	185	9	46	0.0556	0.249	0.193
7	Aurora Police Dept.	151	475	5	97	0.0331	0.204	0.171
8	Phoenix Police Dept.	183	173	0	27	0	0.156	0.156
9	Chicago Police Dept.	168	190	0	29	0	0.153	0.153
10	Tampa Police Dept.	110	91	0	13	0	0.143	0.143
11	Sacramento Police Dept.	152	113	0	13	0	0.115	0.115
12	Austin Police Dept.	166	237	0	27	0	0.114	0.114
13	Miami Police Dept.	494	269	0	29	0	0.108	0.108
14	Anne Arundel Police Dept.	189	134	0	14	0	0.104	0.104
15	Boston Police Dept.	213	224	0	23	0	0.103	0.103
16	Bakersfield Police Dept.	134	160	0	16	0	0.1	0.1
17	Kansas City Police Dept.	269	418	1	37	0.00372	0.0885	0.0848
18	Las Vegas Police Dept.	107	71	0	6	0	0.0845	0.0845
19	Alameda County Sheriff’s Office	179	163	0	12	0	0.0736	0.0736
20	Fort Worth Police Dept.	193	150	0	11	0	0.0733	0.0733
21	Broward County Sheriff’s Office	151	167	0	12	0	0.0719	0.0719
22	Columbus Police Dept.	293	305	0	20	0	0.0656	0.0656
23	Omaha Police Dept.	215	335	0	21	0	0.0627	0.0627
24	Jacksonville Sheriff’s Office	142	162	0	10	0	0.0617	0.0617
25	San Diego County Sheriff’s Dept.	139	133	0	8	0	0.0602	0.0602
26	Hillsborough County Sheriff’s Office	233	264	0	15	0	0.0568	0.0568
27	Oklahoma City Police Dept.	178	223	0	12	0	0.0538	0.0538
28	El Paso County Sheriff’s Office	133	119	0	5	0	0.042	0.042
29	Houston Police Dept.	444	477	4	23	0.00901	0.0482	0.0392
30	Los Angeles County Sheriff’s Dept.	266	211	1	9	0.00376	0.0427	0.0389
31	Milwaukee Police Dept.	424	803	0	29	0	0.0361	0.0361
32	Prince William County Police Dept.	257	337	0	12	0	0.0356	0.0356
33	Baltimore Police Dept.	161	229	0	8	0	0.0349	0.0349
34	Gwinnett County Police Dept.	119	156	0	5	0	0.0321	0.0321
35	Richland County Sheriff’s Dept.	623	398	0	12	0	0.0302	0.0302
36	Palm Beach County Sheriff’s Office	164	206	0	6	0	0.0291	0.0291
37	New York Police Dept.	663	428	0	12	0	0.028	0.028
38	Denver Police Dept.	791	1217	0	22	0	0.0181	0.0181
39	Montgomery County(MD) Police Dept.	177	261	0	4	0	0.0153	0.0153
40	Chesterfield County Police Dept.	118	67	0	1	0	0.0149	0.0149
41	Jefferson County(AL) Sheriff’s Dept.	125	137	0	2	0	0.0146	0.0146
42	D.C. Metropolitan Police Dept.	778	1088	0	15	0	0.0138	0.0138
43	Pierce County Sheriff’s Dept.	153	77	0	1	0	0.013	0.013
44	Wichita Police Dept.	164	81	0	1	0	0.0123	0.0123
45	Harris county Sheriff’s Office	222	192	0	2	0	0.0104	0.0104
46	Jefferson County(CO) Sheriff’s Office	121	203	0	2	0	0.00985	0.00985
47	Fairfax County Police Dept.	366	318	1	3	0.00273	0.00943	0.0067
48	Baltimore County Police Dept.	218	158	0	1	0	0.00633	0.00633
49	Memphis Police Dept.	118	187	0	1	0	0.00535	0.00535
50	Honolulu Police Dept.	195	215	0	1	0	0.00465	0.00465
51	Bernalillo County Sheriff’s Dept.	209	219	0	1	0	0.00457	0.00457
52	Prince George’s County Police Dept.	218	228	0	1	0	0.00439	0.00439
53	Orange County(FL) Sheriff’s Office	575	638	11	14	0.0191	0.0219	0.00281
54	Douglas County(CO) Sheriff’s Office	117	112	0	0	0	0	0
55	Henrico County Police Dept.	143	108	0	0	0	0	0
56	Loudoun County Sheriff’s Office	114	121	0	0	0	0	0
57	Manatee County(FL) Sheriff’s Office	116	128	0	0	0	0	0
58	Montgomery County (TX) Sheriff’s Office	125	104	0	0	0	0	0
59	Pinellas County Police Dept.	223	41	0	0	0	0	0
60	Washington County Sheriff’s Office	126	151	0	0	0	0	0

**Table 10:** Agencies (in the lower-use group) ranked by the increase in posting category 1 (civil unrest related) tweets before and after the killing.

#	Agency name	Total number of tweets pre-killing	Total number of tweets post-killing	Number of C1 tweets pre-killing	Number of C1 tweets post-killing	Percentage of C1 tweets pre-killing	Percentage of C1 tweets post-killing	Percentage change
1	Cleveland Police Dept.	18	75	0	47	0	0.627	0.627
2	Stockton(CA) Police Dept.	35	53	0	33	0	0.623	0.623
3	Anaheim Police Dept.	57	47	0	21	0	0.447	0.447
4	Tulsa Police Dept.	46	101	0	41	0	0.406	0.406
5	Oakland(CA) Police Dept.	65	95	0	28	0	0.295	0.295
6	Louisville Metropolitan Police Dept.	20	41	0	11	0	0.268	0.268
7	Albuquerque Police Dept.	79	49	1	12	0.0127	0.245	0.232
8	Atlanta Police Dept.	33	69	0	16	0	0.232	0.232
9	Metropolitan Nashville Police Dept.	105	118	1	26	0.00952	0.22	0.211
10	Minneapolis Police Dept.	19	48	0	10	0	0.208	0.208
11	Long Beach Police Dept.	41	232	0	46	0	0.198	0.198
12	Detroit Police Dept.	42	71	0	14	0	0.197	0.197
13	Lexington Police Dept.	38	72	0	14	0	0.194	0.194
14	Colorado Springs Police Dept.	85	118	1	24	0.0118	0.203	0.192
15	Riverside(CA) Police Dept.	31	55	0	10	0	0.182	0.182
16	Los Angeles Police Dept.	89	175	2	35	0.0225	0.2	0.178
17	Saint Paul Police Dept.	36	105	0	18	0	0.171	0.171
18	Pittsburgh Bureau of Police	79	128	0	20	0	0.156	0.156
19	Philadelphia Police Dept.	62	130	0	20	0	0.154	0.154
20	Unified (Salt Lake, Utah) Police Dept.	33	17	0	2	0	0.118	0.118
21	Santa Clara County Sheriff’s Office	31	19	0	2	0	0.105	0.105
22	St. Louis County Police Dept.	93	97	0	10	0	0.103	0.103
23	San Francisco Police Dept.	92	101	0	9	0	0.0891	0.0891
24	San Jose Police Dept.	19	27	0	2	0	0.0741	0.0741
25	Franklin County Sheriff’s Office	75	70	0	5	0	0.0714	0.0714
26	San Antonio Police Dept.	21	36	0	2	0	0.0556	0.0556
27	Sacramento County Sheriff’s Office	37	20	0	1	0	0.05	0.05
28	Mesa Police Dept.	101	41	0	2	0	0.0488	0.0488
29	Virginia Beach Police Dept.	41	95	0	4	0	0.0421	0.0421
30	Howard County Police Dept.	50	30	0	1	0	0.0333	0.0333
31	Arlington Police Dept.	88	155	0	5	0	0.0323	0.0323
32	Washoe County Sheriff’s Office	81	131	0	4	0	0.0305	0.0305
33	El Paso Police Dept.	61	73	0	2	0	0.0274	0.0274
34	Tucson Police Dept.	43	37	0	1	0	0.027	0.027
35	Corpus Christi Police Dept.	45	40	0	1	0	0.025	0.025
36	Hennepin County Sheriff’s Office	61	40	0	1	0	0.025	0.025
37	Suffolk County Police Dept.	70	43	0	1	0	0.0233	0.0233
38	St. Louis Police Dept.	81	90	1	3	0.0123	0.0333	0.021
39	Pima County Sheriff’s Dept.	65	170	0	3	0	0.0176	0.0176
40	Ventura County Sheriff’s Office	78	115	0	2	0	0.0174	0.0174
41	Santa Ana Police Dept.	99	120	0	2	0	0.0167	0.0167
42	Lee County(FL) Sheriff’s Office	81	66	0	1	0	0.0152	0.0152
43	Osceola County Sheriff’s Office	70	67	0	1	0	0.0149	0.0149
44	Arapahoe County Sheriff’s Office	66	72	0	1	0	0.0139	0.0139
45	Shelby County(TN) Sheriff’s Office	43	76	0	1	0	0.0132	0.0132
46	Adams County Sheriff’s Office	94	127	0	0	0	0	0
47	Collier County Sheriff’s Office	49	78	0	0	0	0	0
48	Dekalb County Police Dept.	69	71	0	0	0	0	0
49	East Baton Rouge Sheriff’s Office	62	24	0	0	0	0	0
50	Kern County Sheriff’s Dept.	91	75	0	0	0	0	0
51	New Castle County Police Dept.	57	45	0	0	0	0	0
52	Seminole County Sheriff’s Office	33	23	0	0	0	0	0
53	Travis County Sheriff’s Office	20	32	0	0	0	0	0
54	Volusia County Sheriff’s Office	94	71	2	1	0.0213	0.0141	-0.00719
55	Orange County(CA) Sheriff’s Dept.	56	87	3	2	0.0536	0.023	-0.0306

**Table 11:** Agencies (in the higher-use group) ranked by the increase in posting category 7 (community engagement and outreach) tweets before and after the killing.

#	Agency name	Total number of tweets pre-killing	Total number of tweets post-killing	Number of C7 tweets pre-killing	Number of C7 tweets post-killing	Percentage of C7 tweets pre-killing	Percentage of C7 tweets post-killing	Percentage change
1	New York Police Dept.	663	428	179	205	0.27	0.479	0.209
2	Orange County(FL) Sheriff’s Office	575	638	275	385	0.478	0.603	0.125
3	Miami Police Dept.	494	269	163	122	0.33	0.454	0.124
4	Pinellas County Police Dept.	223	41	18	8	0.0807	0.195	0.114
5	Richland County Sheriff’s Dept.	623	398	378	279	0.607	0.701	0.0943
6	Harris county Sheriff’s Office	222	192	77	84	0.347	0.438	0.0907
7	San Diego County Sheriff’s Dept.	139	133	84	89	0.604	0.669	0.0649
8	Baltimore Police Dept.	161	229	31	55	0.193	0.24	0.0476
9	Washington County Sheriff’s Office	126	151	60	79	0.476	0.523	0.047
10	Omaha Police Dept.	215	335	130	215	0.605	0.642	0.0371
11	Wichita Police Dept.	164	81	70	36	0.427	0.444	0.0176
12	Broward County Sheriff’s Office	151	167	115	130	0.762	0.778	0.0169
13	D.C. Metropolitan Police Dept.	778	1088	121	174	0.156	0.16	0.0044
14	Denver Police Dept.	791	1217	38	57	0.048	0.0468	-0.0012
15	Montgomery County(MD) Police Dept.	177	261	27	39	0.153	0.149	-0.00312
16	Memphis Police Dept.	118	187	4	3	0.0339	0.016	-0.0179
17	Jefferson County(AL) Sheriff’s Dept.	125	137	105	112	0.84	0.818	-0.0225
18	Gwinnett County Police Dept.	119	156	41	50	0.345	0.321	-0.024
19	Austin Police Dept.	166	237	39	49	0.235	0.207	-0.0282
20	Oklahoma City Police Dept.	178	223	61	70	0.343	0.314	-0.0288
21	Alameda County Sheriff’s Office	179	163	61	50	0.341	0.307	-0.034
22	Milwaukee Police Dept.	424	803	47	58	0.111	0.0722	-0.0386
23	Honolulu Police Dept.	195	215	48	44	0.246	0.205	-0.0415
24	Prince William County Police Dept.	257	337	127	152	0.494	0.451	-0.0431
25	Kansas City Police Dept.	269	418	115	159	0.428	0.38	-0.0471
26	Houston Police Dept.	444	477	125	111	0.282	0.233	-0.0488
27	Bernalillo County Sheriff’s Dept.	209	219	66	53	0.316	0.242	-0.0738
28	Chicago Police Dept.	168	190	116	117	0.69	0.616	-0.0747
29	Fort Worth Police Dept.	193	150	83	53	0.43	0.353	-0.0767
30	Charlotte-Mecklenburg Police Dept.	195	314	68	85	0.349	0.271	-0.078
31	Bakersfield Police Dept.	134	160	34	27	0.254	0.169	-0.085
32	Columbus Police Dept.	293	305	93	70	0.317	0.23	-0.0879
33	Prince George’s County Police Dept.	218	228	41	22	0.188	0.0965	-0.0916
34	Palm Beach County Sheriff’s Office	164	206	106	114	0.646	0.553	-0.0929
35	Manatee County(FL) Sheriff’s Office	116	128	67	62	0.578	0.484	-0.0932
36	Jefferson County(CO) Sheriff’s Office	121	203	55	73	0.455	0.36	-0.0949
37	Loudoun County Sheriff’s Office	114	121	76	66	0.667	0.545	-0.121
38	Boston Police Dept.	213	224	88	65	0.413	0.29	-0.123
39	Raleigh Police Dept.	162	185	52	36	0.321	0.195	-0.126
40	Los Angeles County Sheriff’s Dept.	266	211	132	78	0.496	0.37	-0.127
41	El Paso County Sheriff’s Office	133	119	55	34	0.414	0.286	-0.128
42	Montgomery County (TX) Sheriff’s Office	125	104	75	49	0.6	0.471	-0.129
43	Seattle Police Dept.	117	226	35	37	0.299	0.164	-0.135
44	Fairfax County Police Dept.	366	318	121	60	0.331	0.189	-0.142
45	Aurora Police Dept.	151	475	34	35	0.225	0.0737	-0.151
46	Douglas County(CO) Sheriff’s Office	117	112	79	55	0.675	0.491	-0.184
47	Jacksonville Sheriff’s Office	142	162	51	27	0.359	0.167	-0.192
48	Las Vegas Police Dept.	107	71	63	28	0.589	0.394	-0.194
49	Chesterfield County Police Dept.	118	67	69	26	0.585	0.388	-0.197
50	Anne Arundel Police Dept.	189	134	111	52	0.587	0.388	-0.199
51	Tampa Police Dept.	110	91	65	35	0.591	0.385	-0.206
52	Hillsborough County Sheriff’s Office	233	264	157	122	0.674	0.462	-0.212
53	Baltimore County Police Dept.	218	158	100	36	0.459	0.228	-0.231
54	Portland Police Dept.	303	900	105	100	0.347	0.111	-0.235
55	San Diego Police Dept.	200	191	130	76	0.65	0.398	-0.252
56	Henrico County Police Dept.	143	108	59	15	0.413	0.139	-0.274
57	Dallas Police Dept.	425	491	228	123	0.536	0.251	-0.286
58	Sacramento Police Dept.	152	113	105	43	0.691	0.381	-0.31
59	Pierce County Sheriff’s Dept.	153	77	112	28	0.732	0.364	-0.368
60	Phoenix Police Dept.	183	173	137	64	0.749	0.37	-0.379

**Table 12:** Agencies (in the lower-use group) ranked by the increase in posting category 7 (community engagement and outreach) tweets before and after the killing.

#	Agency name	Total number of tweets pre-killing	Total number of tweets post-killing	Number of C7 tweets pre-killing	Number of C7 tweets post-killing	Percentage of C7 tweets pre-killing	Percentage of C7 tweets post-killing	Percentage change
1	San Jose Police Dept.	19	27	13	22	0.684	0.815	0.131
2	Santa Clara County Sheriff’s Office	31	19	18	13	0.581	0.684	0.104
3	Hennepin County Sheriff’s Office	61	40	43	32	0.705	0.8	0.0951
4	Osceola County Sheriff’s Office	70	67	30	32	0.429	0.478	0.049
5	Suffolk County Police Dept.	70	43	51	33	0.729	0.767	0.0389
6	Arapahoe County Sheriff’s Office	66	72	47	54	0.712	0.75	0.0379
7	Ventura County Sheriff’s Office	78	115	19	32	0.244	0.278	0.0347
8	Unified (Salt Lake, Utah) Police Dept.	33	17	15	8	0.455	0.471	0.016
9	Collier County Sheriff’s Office	49	78	17	28	0.347	0.359	0.012
10	Kern County Sheriff’s Dept.	91	75	39	33	0.429	0.44	0.0114
11	Lee County(FL) Sheriff’s Office	81	66	64	52	0.79	0.788	-0.00224
12	Orange County(CA) Sheriff’s Dept.	56	87	33	51	0.589	0.586	-0.00308
13	Dekalb County Police Dept.	69	71	24	23	0.348	0.324	-0.0239
14	Saint Paul Police Dept.	36	105	11	29	0.306	0.276	-0.0294
15	Pima County Sheriff’s Dept.	65	170	22	51	0.338	0.3	-0.0385
16	Philadelphia Police Dept.	62	130	7	9	0.113	0.0692	-0.0437
17	Arlington Police Dept.	88	155	57	93	0.648	0.6	-0.0477
18	New Castle County Police Dept.	57	45	17	11	0.298	0.244	-0.0538
19	Adams County Sheriff’s Office	94	127	66	82	0.702	0.646	-0.0565
20	Riverside(CA) Police Dept.	31	55	12	18	0.387	0.327	-0.0598
21	East Baton Rouge Sheriff’s Office	62	24	33	11	0.532	0.458	-0.0739
22	Atlanta Police Dept.	33	69	21	38	0.636	0.551	-0.0856
23	Franklin County Sheriff’s Office	75	70	43	33	0.573	0.471	-0.102
24	Minneapolis Police Dept.	19	48	10	20	0.526	0.417	-0.11
25	San Francisco Police Dept.	92	101	54	48	0.587	0.475	-0.112
26	Howard County Police Dept.	50	30	21	9	0.42	0.3	-0.12
27	Sacramento County Sheriff’s Office	37	20	12	4	0.324	0.2	-0.124
28	Los Angeles Police Dept.	89	175	40	56	0.449	0.32	-0.129
29	Santa Ana Police Dept.	99	120	53	48	0.535	0.4	-0.135
30	Volusia County Sheriff’s Office	94	71	37	18	0.394	0.254	-0.14
31	San Antonio Police Dept.	21	36	10	12	0.476	0.333	-0.143
32	El Paso Police Dept.	61	73	34	29	0.557	0.397	-0.16
33	Washoe County Sheriff’s Office	81	131	62	79	0.765	0.603	-0.162
34	Oakland(CA) Police Dept.	65	95	38	40	0.585	0.421	-0.164
35	Shelby County(TN) Sheriff’s Office	43	76	15	14	0.349	0.184	-0.165
36	Virginia Beach Police Dept.	41	95	14	16	0.341	0.168	-0.173
37	St. Louis County Police Dept.	93	97	67	53	0.72	0.546	-0.174
38	Detroit Police Dept.	42	71	27	33	0.643	0.465	-0.178
39	Metropolitan Nashville Police Dept.	105	118	56	40	0.533	0.339	-0.194
40	Pittsburgh Bureau of Police	79	128	33	28	0.418	0.219	-0.199
41	Tulsa Police Dept.	46	101	22	27	0.478	0.267	-0.211
42	Tucson Police Dept.	43	37	20	9	0.465	0.243	-0.222
43	Albuquerque Police Dept.	79	49	42	15	0.532	0.306	-0.226
44	Seminole County Sheriff’s Office	33	23	22	10	0.667	0.435	-0.232
45	Louisville Metropolitan Police Dept.	20	41	10	10	0.5	0.244	-0.256
46	Corpus Christi Police Dept.	45	40	27	13	0.6	0.325	-0.275
47	Lexington Police Dept.	38	72	22	18	0.579	0.25	-0.329
48	St. Louis Police Dept.	81	90	40	14	0.494	0.156	-0.338
49	Mesa Police Dept.	101	41	72	15	0.713	0.366	-0.347
50	Stockton(CA) Police Dept.	35	53	15	4	0.429	0.0755	-0.353
51	Travis County Sheriff’s Office	20	32	15	12	0.75	0.375	-0.375
52	Colorado Springs Police Dept.	85	118	54	30	0.635	0.254	-0.381
53	Anaheim Police Dept.	57	47	35	8	0.614	0.17	-0.444
54	Cleveland Police Dept.	18	75	12	10	0.667	0.133	-0.533
55	Long Beach Police Dept.	41	232	28	21	0.683	0.0905	-0.592

Tables 3 and 4 rank agencies in the higher- and lower-use group based on how substantially they increased or decreased their tweeting practices pre- and post-killing. For instance, the Aurora Police Department (ranked #1 in the higher-use group for this dimension) posted 152 tweets in the pre-killing period and 477 tweets in the post-killing period. The average number of posts per day was 2.76 (152 tweets/55 days) and 7.12 (477 tweets/67 days) pre- and post-killing. This translates to a raw frequency change of 4.36 (7.12–2.76) and a percentage change of 158% (7.12/2.76–1). Tables 5 and 6 rank the agencies based on the increase or decrease in the average number of favorites they received per tweet pre- and post-killing. For instance, tweets from the Venture County Sheriff’s Office (ranked #1 in the lower-use group for this dimension), on average, received 11.5 favorites in the pre-killing period and 139 favorites in the post-killing period, a raw favorite change of 127.5 (139–11.5) and a percentage change of 1117% (139/11.5–1). In a similar vein, Tables 7 and 8 rank the agencies based on the increase or decrease in the average number of retweets they received per tweet pre- and post-killing.

Moreover, Tables 9 and 10 rank the agencies based on the increase or decrease in posting civil unrest related tweets pre- and post-killing. For instance, the Portland Police Department (ranked #1 in the higher-use group for this dimension) posted a total of 303 tweets in the pre-killing period and none of the tweets were civil unrest related. Yet, in the post-killing period, they posted a total of 900 tweets, 535 of which were civil unrest related. Thus, the proportion change was 0.594 (535/900–0/303). Tables 11 and 12 rank the agencies based on the increase or decrease in posting community engagement and outreach tweets pre- and post-killing. For example, the NYPD (ranked #1 in the higher-use group for this dimension) posted a total of 663 tweets in the pre-killing period and 179 of the tweets were for community engagement and outreach purpose; in the post-killing period, they posted a total of 428 tweets, 205 of which were for community engagement and outreach purpose. Thus, the proportion change was 0.209 (205/428–179/663).

Finally, we combined the five dimensions above (Tables 3 through 12) and constructed an overall ranking to gauge which law enforcement agencies may have more effectively reached and engaged (or governed) citizens through Twitter. To offer a straightforward understanding, the five dimensions were assumed equal weights in our attempt and their corresponding ranks were averaged to derive an overall ranking. Tables 13 and 14 illustrate the overall rankings for the higher- and lower-use group. For example, the Charlotte-Mecklenburg Police Department (in the higher-use group) ranked 5^th^ in posting more tweets per day, 3^rd^ in receiving more favorites per tweet, 4^th^ in receiving more retweets per tweet, 5^th^ in posting a higher percentage of civil unrest related tweets, and 30^th^ in posting a higher percentage of community engagement and outreach tweets before vs. after the killing. The mean equaled 9.4 ((5+3+4+5+30)/5) across the five rankings and placed the Charlotte-Mecklenburg Police Department 1^st^ in the overall rank. It is worth noting that none of the police departments ranked (very) high on all five dimensions. In particular, if they increased their posting of civil unrest related tweets in the post-killing period, they were likely to reduce posting community engagement and outreach tweets.

**Table 13:** Agencies (in the higher-use group) ranked by the five dimensions combined.

#	Agency name	Rank A	Rank B	Rank C	Rank D	Rank E	Average rank	Overall rank
1	Charlotte-Mecklenburg Police Dept.	5	3	4	5	30	9.4	1
2	Seattle Police Dept.	4	2	2	3	43	10.8	2
3	Portland Police Dept.	2	1	1	1	54	11.8	3
4	Austin Police Dept.	12	6	11	12	19	12	4
5	Milwaukee Police Dept.	3	8	15	31	22	15.8	5
6	Omaha Police Dept.	8	15	24	23	10	16	6
7	Baltimore Police Dept.	13	20	12	33	8	17.2	7
8	Oklahoma City Police Dept.	18	12	17	27	20	18.8	8
9	Denver Police Dept.	11	18	18	38	14	19.8	9
10	Aurora Police Dept.	1	31	16	7	45	20	10
11	Alameda County Sheriff’s Office	40	13	9	19	21	20.4	11
12	Columbus Police Dept.	33	9	7	22	32	20.6	12
13	Miami Police Dept.	57	17	13	13	3	20.6	13
14	Dallas Police Dept.	22	11	14	2	57	21.2	14
15	D.C. Metropolitan Police Dept.	14	16	27	42	13	22.4	15
16	San Diego Police Dept.	43	5	5	4	55	22.4	16
17	Chicago Police Dept.	25	29	25	9	28	23.2	17
18	Tampa Police Dept.	45	4	6	10	51	23.2	18
19	New York Police Dept.	54	14	10	37	1	23.2	19
20	Wichita Police Dept.	59	10	3	44	11	25.4	20
21	Gwinnett County Police Dept.	17	21	38	34	18	25.6	21
22	Kansas City Police Dept.	9	34	46	17	25	26.2	22
23	Memphis Police Dept.	6	25	39	49	16	27	23
24	Boston Police Dept.	34	22	31	15	38	28	24
25	Hillsborough County Sheriff’s Office	26	19	19	26	52	28.4	25
26	Fort Worth Police Dept.	47	27	23	20	29	29.2	26
27	Houston Police Dept.	30	26	36	29	26	29.4	27
28	Phoenix Police Dept.	39	24	20	8	60	30.2	28
29	Sacramento Police Dept.	51	23	8	11	58	30.2	29
30	San Diego County Sheriff’s Dept.	38	32	54	25	7	31.2	30
31	Pinellas County Police Dept.	60	7	29	59	4	31.8	31
32	Jefferson County(AL) Sheriff’s Dept.	24	28	50	41	17	32	32
33	Prince William County Police Dept.	16	41	49	32	24	32.4	33
34	Montgomery County(MD) Police Dept.	10	54	45	39	15	32.6	34
35	Raleigh Police Dept.	15	53	53	6	39	33.2	35
36	Richland County Sheriff’s Dept.	55	39	32	35	5	33.2	36
37	Palm Beach County Sheriff’s Office	21	44	34	36	34	33.8	37
38	Bakersfield Police Dept.	20	47	56	16	31	34	38
39	Las Vegas Police Dept.	48	35	22	18	48	34.2	39
40	Orange County(FL) Sheriff’s Office	27	45	51	53	2	35.6	40
41	Harris county Sheriff’s Office	44	42	42	45	6	35.8	41
42	Jefferson County(CO) Sheriff’s Office	7	49	44	46	36	36.4	42
43	Broward County Sheriff’s Office	37	57	57	21	12	36.8	43
44	Jacksonville Sheriff’s Office	23	50	41	24	47	37	44
45	Bernalillo County Sheriff’s Dept.	32	55	30	51	27	39	45
46	Fairfax County Police Dept.	42	37	26	47	44	39.2	46
47	Los Angeles County Sheriff’s Dept.	49	40	37	30	40	39.2	47
48	Chesterfield County Police Dept.	56	30	21	40	49	39.2	48
49	Douglas County(CO) Sheriff’s Office	36	33	28	54	46	39.4	49
50	Washington County Sheriff’s Office	19	58	52	60	9	39.6	50
51	Prince George’s County Police Dept.	35	36	47	52	33	40.6	51
52	Honolulu Police Dept.	29	43	59	50	23	40.8	52
53	Loudoun County Sheriff’s Office	31	52	35	56	37	42.2	53
54	Manatee County(FL) Sheriff’s Office	28	51	48	57	35	43.8	54
55	El Paso County Sheriff’s Office	41	56	55	28	41	44.2	55
56	Baltimore County Police Dept.	53	46	33	48	53	46.6	56
57	Montgomery County (TX) Sheriff’s Office	46	48	40	58	42	46.8	57
58	Anne Arundel Police Dept.	52	59	60	14	50	47	58
59	Pierce County Sheriff’s Dept.	58	38	43	43	59	48.2	59
60	Henrico County Police Dept.	50	60	58	55	56	55.8	60

**Table 14:** Agencies (in the lower-use group) ranked by the five dimensions combined.

#	Agency name	Rank A	Rank B	Rank C	Rank D	Rank E	Average rank	Overall rank
1	Saint Paul Police Dept.	3	2	3	17	14	7.8	1
2	Minneapolis Police Dept.	5	4	9	10	24	10.4	2
3	Atlanta Police Dept.	7	3	23	8	22	12.6	3
4	Louisville Metropolitan Police Dept.	8	5	2	6	45	13.2	4
5	Philadelphia Police Dept.	9	7	15	19	16	13.2	5
6	San Jose Police Dept.	19	15	13	24	1	14.4	6
7	Ventura County Sheriff’s Office	24	1	1	40	7	14.6	7
8	Tulsa Police Dept.	10	13	11	4	41	15.8	8
9	Cleveland Police Dept.	2	12	18	1	54	17.4	9
10	Unified (Salt Lake, Utah) Police Dept.	53	6	7	20	8	18.8	10
11	Pima County Sheriff’s Dept.	4	16	21	39	15	19	11
12	Lexington Police Dept.	12	10	16	13	47	19.6	12
13	Stockton(CA) Police Dept.	25	9	14	2	50	20	13
14	Metropolitan Nashville Police Dept.	31	14	12	9	39	21	14
15	Oakland(CA) Police Dept.	26	23	19	5	34	21.4	15
16	Detroit Police Dept.	17	27	17	12	38	22.2	16
17	Anaheim Police Dept.	43	8	6	3	53	22.6	17
18	Los Angeles Police Dept.	11	26	34	16	28	23	18
19	San Antonio Police Dept.	13	20	29	26	31	23.8	19
20	St. Louis County Police Dept.	35	21	8	22	37	24.6	20
21	Riverside(CA) Police Dept.	14	25	52	15	20	25.2	21
22	Albuquerque Police Dept.	49	18	10	7	43	25.4	22
23	Osceola County Sheriff’s Office	37	19	25	43	4	25.6	23
24	Santa Clara County Sheriff’s Office	51	28	27	21	2	25.8	24
25	St. Louis Police Dept.	34	11	4	38	48	27	25
26	Long Beach Police Dept.	1	31	39	11	55	27.4	26
27	Colorado Springs Police Dept.	27	24	20	14	52	27.4	27
28	Hennepin County Sheriff’s Office	47	29	24	36	3	27.8	28
29	Virginia Beach Police Dept.	6	40	33	29	36	28.8	29
30	San Francisco Police Dept.	32	33	35	23	25	29.6	30
31	Arlington Police Dept.	15	39	47	31	17	29.8	31
32	Sacramento County Sheriff’s Office	52	38	5	27	27	29.8	32
33	Arapahoe County Sheriff’s Office	33	37	30	44	6	30	33
34	Howard County Police Dept.	50	17	28	30	26	30.2	34
35	Franklin County Sheriff’s Office	38	30	36	25	23	30.4	35
36	Dekalb County Police Dept.	36	34	22	48	13	30.6	36
37	Santa Ana Police Dept.	30	32	32	41	29	32.8	37
38	Collier County Sheriff’s Office	22	43	44	47	9	33	38
39	Pittsburgh Bureau of Police	20	53	41	18	40	34.4	39
40	Suffolk County Police Dept.	48	42	42	37	5	34.8	40
41	El Paso Police Dept.	29	46	37	33	32	35.4	41
42	Washoe County Sheriff’s Office	16	50	48	32	33	35.8	42
43	Shelby County(TN) Sheriff’s Office	18	41	40	45	35	35.8	43
44	Orange County(CA) Sheriff’s Dept.	23	44	49	55	12	36.6	44
45	Lee County(FL) Sheriff’s Office	42	48	45	42	11	37.6	45
46	Mesa Police Dept.	54	35	26	28	49	38.4	46
47	Corpus Christi Police Dept.	39	36	38	35	46	38.8	47
48	Kern County Sheriff’s Dept.	41	47	46	50	10	38.8	48
49	Adams County Sheriff’s Office	28	51	51	46	19	39	49
50	Seminole County Sheriff’s Office	46	22	31	52	44	39	50
51	Tucson Police Dept.	40	49	50	34	42	43	51
52	Volusia County Sheriff’s Office	45	45	43	54	30	43.4	52
53	New Castle County Police Dept.	44	55	54	51	18	44.4	53
54	Travis County Sheriff’s Office	21	52	55	53	51	46.4	54
55	East Baton Rouge Sheriff’s Office	55	54	53	49	21	46.4	55

## Discussion

The current study explored police tweeting practices in a sample of 115 large agencies in the U.S., approximately two months before and after the killing of George Floyd that sparked the nationwide protests directed at the police in 2020.

In line with image repair theory, our analyses provided insights into the specific activities police agencies engaged in on social media in response to image damage and public reactions to those activities. Specifically, law enforcement agencies tweeted more frequently in the immediate aftermath of the killing and posted an increased number of civil-unrest related tweets. Police also continued to communicate case updates, perhaps directing public attention to their traditional role and responsibilities in fighting crime and maintaining order. On the other end, the public (at least those who were exposed to police departments tweets) showed a greater interest in engaging with law enforcement agencies. The rate at which the public favorited or retweeted a police tweet went up significantly following the George Floyd incident and stayed higher than before throughout the rest of the study period. Changes in the focal issues of police tweets (and potentially an increased attention to police behavior) may partially explain the increases in favorites and retweets received per tweet despite the police being in a legitimacy crisis. In particular, police tweets related to civil unrest, on average, received public reactions between twice and 20 times more than those of other categories of tweets received. By channeling and amplifying public energy towards this issue, social media provides opportunities for law enforcement to respond, engage, and rectify any misinformation with high efficiencies.

It may not be surprising that agencies that had the largest increases in public reactions (i.e., average favorite and retweet counts) or protest-related posts were from cities that saw major protest and riot activities, which aligns with the image repair thesis. However, we cannot conclude whether these increases were results of police engagement efforts aiming to genuinely improve police-community relations or socialization/legitimation efforts of reputation management. For instance, category 1 (or civil unrest related) tweets covered such topics as operational responses to the protest, crime and violence committed during the riots, challenges to racial justice in policing, and injuries and hostilities to police. These subcategories tap genuine concerns about racial injustice in the U.S. but also governance of citizens. We aimed to further distinguish subcategories within a focal issue (e.g., our labeled data included such information). Yet, the very low frequency of some subcategories in the labeled data and the limits of our prediction models prohibited us from pursuing this route. Nonetheless, it is clear that individual police agencies varied vastly in their social media usage. Not every agency was actively using Twitter to reach and engage or govern people. Twenty-four of the initial 139 large police agencies identified did not have a regular presence on Twitter. The final sample of 115 large police agencies also demonstrated tremendous variability in how often they tweeted, the types of tweets they tended to post, and public reactions to their social media content both at the baseline level and during the protest. Examining only a handful of agencies or a particular dimension of social media usage seems unlikely to provide a complete picture of police behaviors and citizen interactions on Twitter.

We contributed to the literature of police use of social media by creating a single indicator that combined measures of changes in the quantity of tweets, composition of tweets, and public responses to those tweets. While prior studies on police use of social media have looked at the number and types of police posts and the diffusion of those posts individually, few engaged in efforts to show where law enforcement agencies are relative to each other with respect to different sub-metrics and the overall standing of Twitter use. We controlled for baseline social media activity levels by separating our sample into higher versus lower activity agencies, thus adjusting for potential influences of agency-level factors on social media usage and its changes (e.g., agency size and jurisdiction population). Our combined indicator may serve as a useful first step toward cultivating responsible and effective use of social media by police. That said, the current study represents a preliminary effort at quantifying and understanding police social media usage in the context of the George Floyd protests and does not represent a comprehensive measurement of police performance on social media overall.

Given the great variability in police social media usage observed in our study and different possible interpretations of these efforts (e.g., engagement vs. socialization/legitimation), we do not recommend the deactivation of all police Twitter accounts as suggested by some [13]. Instead, we suggest a few avenues for future research (and practices) on responsible and effective use of social media by police, while pointing out the challenges associated with such inquiries.

First, future studies should explore why (and how) changes in police social media usage occur before and after a major social event. A few important challenges remain. We are uncertain of who are interacting with police on social media, which has important implications to its impact on police legitimacy or police-community relations. Much of the legitimacy crisis reflects ongoing frictions between police and disadvantaged/minority communities, the dynamic of which may not be captured by examining the overall responses received by police tweets. For instance, rather than reflecting improvements in community engagement activities or citizen trust, the increases in favorites and retweets of police-generated content might reflect more active reactions from a pre-existing pro-police audience. Police engagement efforts, however, are most needed towards minority and disadvantaged groups who are regularly contacted by law enforcement agencies. Whether and through what strategies police social media usage can target, reach, and respond to those groups would largely determine the efficacy of online police-community interactions, especially in repairing harm and (re)gaining trust. Otherwise, police communications on Twitter may not be fundamentally different from traditional means of communication and mainly fulfill a function of socialization/legitimation or appeal to those who already endorse police value and activities.

In addition, it is necessary to further investigate the detailed content generated by police on social media. While categorization, as in our case, is helpful in understanding shifts in general directions of police social media usage, topic modeling in natural language processing may uncover themes from a large corpus of tweets and assign individual tweets to different themes, better illustrating police motives for social media usage [42, 43]. Adopting computer-assisted techniques to analyze (at a large scale) hyperlinks, images, and videos contained in police tweets should further improve our understanding of police social media usage [44]. Moreover, it would be helpful to investigate what organizational characteristics are associated with agency-level police motives for social media usage and adjustments after a major challenge.

Second, future studies should assess the impact of police social media usage on other performance measures, including public receptivity, police legitimacy and trust, crime investigation and clearance rate, community informal social control, among others. Such undertaking is challenging given the nature of these inquiries and the data needed for answering these questions yet important. Citizens’ experiences with the police affect their overall assessment of the police, but the vast majority of the American public do not have face-to-face contact with a police officer in any given year [45, 46]. The extension from physical interaction with the police to social media platforms is worth further investigation.

Of note, although some consider liking and reposting behaviors less engaging or dialogical than “real” engagement activities such as community meetings, police-community collaborations, and joint problem-solving efforts, metric-driven engagement has an important meaning in and of itself in an algorithmic environment of social media in which information is curated and disseminated based on their relative popularity. Recognizing the limitations (e.g., ambiguous motives of police social media usage and messages not necessarily reaching targeted groups), scholars have argued that these metrics should be used to guide the development of social media strategies of law enforcement agencies [47], similar to how favorites and retweets are commonly used as indicators of success of a marketing strategy in the private industry. That said, agencies should be cautious not to seek reactions by posting content simply to appeal to their audiences. Authenticity and communicating negative but honest messages have been found to be key to maintaining police credibility on social media [15]. This helps explain the findings from our sentiment analysis. Police-generated social media content exuded greater negative than positive emotions following the George Floyd incident, but public reactions (i.e., average favorite and retweet counts per tweet) also went up tremendously during this time.

The study has limitations. First, police agencies may use social media platforms other than Twitter (e.g., Facebook or Nextdoor) to reach and engage (or govern) people during the same study period. Second, we did not explicitly investigate two-way police-citizen interactions on Twitter. Official police agency Twitter accounts often replied to other non-public Twitter accounts (e.g., a police chief’s account or police precinct account). Given the scale of the current study, manually checking each replying tweet was not feasible. Thus, we could not accurately assess the proportion of two-way police-citizen interactions on Twitter. Our preliminary check (excluding self-replying tweets) indicated that approximately 13% of all included 38,701 tweets were replies and that there were great variabilities in the proportion (e.g., over half of the Denver Police Departments tweets during the study period were replies, whereas several police agencies did not post any replying tweets during the same period of time) and the way official police agency Twitter accounts posted replying tweets. Additionally, we only analyzed the text content of police tweets, yet image or video content (also URLs) may meaningfully affect public reactions to police tweets. Moreover, retweeting does not necessarily reflect agreement with original content (e.g., retweets with users’ own negative reactions). In this sense, our findings show increased public participation in dialogues on public safety and social justice issues, not necessarily increased support for police-generated content online. Third, our classification algorithm was not perfect, but its accuracy was acceptable for our purpose. Fourth, the study examined police departments tweets approximately two months before and after the killing. Adjustments of Twitter usage made by police agencies may take longer to carry out. The scale and intensity of protests (and disruptions) at different jurisdictions may also affect how local police agencies adjust their social media presence, which we could not explicitly study. Future research should also explore geographic and political influences on police use of social media. Finally, given our focus on large police departments in the U.S., the results may not be generalizable to smaller agencies or agencies in other countries in their use of social media during a social crisis event.

## Conclusion

The utility of social media in policing and public governance remains an understudied area, where case studies and qualitative evidence predominate. Through examining Twitter usage by 115 large U.S. police agencies following a major legitimacy crisis, we conclude that police reacted to the George Floyd incident on social media and that the public paid attention to and seemingly held positive attitudes toward those changes. Police agencies in our sample tweeted more frequently following the killing of George Floyd and posted more tweets related to civil unrest as well as case updates. These tweets received greater public reaction (through favorites and retweets), which persisted throughout the study period.

Nonetheless, a great variability emerged across agencies in their responses on social media (e.g., different rates and focuses of use), and the motives for the observed changes pre- and post-event were inconclusive. Future efforts are called for to address the limitations and ambiguities uncovered by this study about police use of social media (e.g., characteristics of those who interact with police on social media, communications that go beyond favorites and retweets, and police behaviors on social media platforms other than Twitter), and to find ways for police to responsibly and effectively utilize various communication platforms in the era of “big data”. For instance, a guideline or protocol of best practices for police social media usage may be developed and made public for comments prior to its approval and implementation, through which “selective transparency” may be curbed.

## Supporting information

S1 Table A complete list of the 115 law enforcement agencies included in the study. (DOCX)

S2 Table Detailed categorization scheme used in the study. (DOCX)

S3 Table Random forest and multiclass boosted trees classifier. (DOCX)

S4 Table Exemplary tweets illustrating sentence-level pleasant or attractive vs. unpleasant or aversive emotion. (DOCX)

S5 Table Supplementary details about the results of the multiclass random forest classifier. (DOCX)
