# Supplementary material for: Reaching and engaging people: Analyzing tweeting practices of large U.S. police departments pre- and post- the killing of George Floyd
Source: PLoS One. 2022 Jul 14;17(7):e0269288. doi: 10.1371/journal.pone.0269288 (PMC9282545; doi:10.1371/journal.pone.0269288)
Supplement: S1 Table — (DOCX) [file pone.0269288.s001.docx]

**Table 1S**. A complete list of the 115 law enforcement agencies included in the study

| **#** | **Agency name** | **Number of tweets** |
| --- | --- | --- |
| 1 | Denver Police Dept. | 2106 |
| 2 | D.C. Metropolitan Police Dept. | 1867 |
| 3 | Milwaukee Police Dept. | 1240 |
| 4 | Portland Police Dept. | 1220 |
| 5 | Orange County(FL) Sheriff's Office | 1214 |
| 6 | Richland County Sheriff's Dept. | 1130 |
| 7 | New York Police Dept. | 1095 |
| 8 | Houston Police Dept. | 938 |
| 9 | Dallas Police Dept. | 918 |
| 10 | Miami Police Dept. | 765 |
| 11 | Kansas City Police Dept. | 701 |
| 12 | Fairfax County Police Dept. | 685 |
| 13 | Aurora Police Dept. | 629 |
| 14 | Columbus Police Dept. | 607 |
| 15 | Prince William County Police Dept. | 595 |
| 16 | Omaha Police Dept. | 557 |
| 17 | Charlotte-Mecklenburg Police Dept. | 521 |
| 18 | Hillsborough County Sheriff's Office | 497 |
| 19 | Los Angeles County Sheriff's Dept. | 478 |
| 20 | Montgomery County(MD) Police Dept. | 453 |
| 21 | Prince George's County Police Dept. | 448 |
| 22 | Boston Police Dept. | 438 |
| 23 | Bernalillo County Sheriff's Dept. | 431 |
| 24 | San Diego Police Dept. | 421 |
| 25 | Harris county Sheriff's Office | 416 |
| 26 | Austin Police Dept. | 411 |
| 27 | Honolulu Police Dept. | 411 |
| 28 | Oklahoma City Police Dept. | 406 |
| 29 | Raleigh Police Dept. | 399 |
| 30 | Baltimore Police Dept. | 395 |
| 31 | Palm Beach County Sheriff's Office | 392 |
| 32 | Baltimore County Police Dept. | 380 |
| 33 | Chicago Police Dept. | 359 |
| 34 | Fort Worth Police Dept. | 357 |
| 35 | Phoenix Police Dept. | 357 |
| 36 | Alameda County Sheriff's Office | 349 |
| 37 | Seattle Police Dept. | 348 |
| 38 | Anne Arundel Police Dept. | 342 |
| 39 | Broward County Sheriff's Office | 341 |
| 40 | Jefferson County(CO) Sheriff's Office | 339 |
| 41 | Memphis Police Dept. | 309 |
| 42 | Jacksonville Sheriff's Office | 307 |
| 43 | Bakersfield Police Dept. | 296 |
| 44 | Washington County Sheriff's Office | 283 |
| 45 | Gwinnett County Police Dept. | 277 |
| 46 | Long Beach Police Dept. | 274 |
| 47 | Los Angeles Police Dept. | 272 |
| 48 | San Diego County Sheriff's Dept. | 272 |
| 49 | Jefferson County(AL) Sheriff's Dept. | 270 |
| 50 | Pinellas County Police Dept. | 269 |
| 51 | Sacramento Police Dept. | 267 |
| 52 | Henrico County Police Dept. | 259 |
| 53 | El Paso County Sheriff's Office | 254 |
| 54 | Wichita Police Dept. | 247 |
| 55 | Manatee County(FL) Sheriff's Office | 244 |
| 56 | Arlington Police Dept. | 243 |
| 57 | Pima County Sheriff's Dept. | 239 |
| 58 | Loudoun County Sheriff’s Office | 235 |
| 59 | Douglas County(CO) Sheriff's Office | 233 |
| 60 | Adams County Sheriff's Office | 231 |
| 61 | Pierce County Sheriff's Dept. | 231 |
| 62 | Montgomery County (TX) Sheriff's Office | 229 |
| 63 | Metropolitan Nashville Police Dept. | 223 |
| 64 | Santa Ana Police Dept. | 221 |
| 65 | Washoe County Sheriff's Office | 221 |
| 66 | Pittsburgh Bureau of Police | 208 |
| 67 | Tampa Police Dept. | 204 |
| 68 | Colorado Springs Police Dept. | 203 |
| 69 | Las Vegas Police Dept. | 202 |
| 70 | Ventura County Sheriff's Office | 195 |
| 71 | San Francisco Police Dept. | 193 |
| 72 | Philadelphia Police Dept. | 192 |
| 73 | St. Louis County Police Dept. | 192 |
| 74 | Chesterfield County Police Dept. | 186 |
| 75 | St. Louis Police Dept. | 174 |
| 76 | Kern County Sheriff's Dept. | 166 |
| 77 | Volusia County Sheriff's Office | 166 |
| 78 | Oakland(CA) Police Dept. | 162 |
| 79 | Mesa Police Dept. | 154 |
| 80 | Tulsa Police Dept. | 154 |
| 81 | Dekalb County Police Dept. | 150 |
| 82 | Virginia Beach Police Dept. | 149 |
| 83 | Lee County(FL) Sheriff's Office | 147 |
| 84 | El Paso Police Dept. | 146 |
| 85 | Franklin County Sheriff’s Office | 146 |
| 86 | Orange County(CA) Sheriff's Dept. | 146 |
| 87 | Saint Paul Police Dept. | 145 |
| 88 | Arapahoe County Sheriff's Office | 141 |
| 89 | Osceola County Sheriff's Office | 137 |
| 90 | Suffolk County Police Dept. | 131 |
| 91 | Albuquerque Police Dept. | 128 |
| 92 | Collier County Sheriff's Office | 128 |
| 93 | Shelby County(TN) Sheriff's Office | 121 |
| 94 | Detroit Police Dept. | 117 |
| 95 | Anaheim Police Dept. | 112 |
| 96 | Lexington Police Dept. | 111 |
| 97 | Atlanta Police Dept. | 104 |
| 98 | New Castle County Police Dept. | 104 |
| 99 | Hennepin County Sheriff's Office | 101 |
| 100 | Cleveland Police Dept. | 97 |
| 101 | Stockton(CA) Police Dept. | 89 |
| 102 | Corpus Christi Police Dept. | 87 |
| 103 | Riverside(CA) Police Dept. | 87 |
| 104 | East Baton Rouge Sheriff's Office | 86 |
| 105 | Howard County Police Dept. | 80 |
| 106 | Tucson Police Dept. | 80 |
| 107 | Minneapolis Police Dept. | 67 |
| 108 | Louisville Metropolitan Police Dept. | 66 |
| 109 | San Antonio Police Dept. | 59 |
| 110 | Sacramento County Sheriff's Office | 58 |
| 111 | San Jose Police Dept. | 56 |
| 112 | Seminole County Sheriff's Office | 56 |
| 113 | Unified (Salt Lake, Utah) Police Dept. | 53 |
| 114 | Travis County Sheriff's Office | 52 |
| 115 | Santa Clara County Sheriff's Office | 51 |
