# Supplementary material for: Reaching and engaging people: Analyzing tweeting practices of large U.S. police departments pre- and post- the killing of George Floyd
Source: PLoS One. 2022 Jul 14;17(7):e0269288. doi: 10.1371/journal.pone.0269288 (PMC9282545; doi:10.1371/journal.pone.0269288)
Supplement: S2 Table — (DOCX) [file pone.0269288.s002.docx]

**Table 2S**. Detailed categorization scheme used in the study

| **Main categories** | **Descriptions** |
| --- | --- |
| **Civil unrest related**  **(focal issue #1)** | General information sharing and updates (including traffic);  Issues related to racial injustice;  Protest-related crime and violence issues;  Police use of force;  Injuries and hostilities to police |
| **COVID-19 related**  **(focal issue #2)** | Police responses to COVID-19 or police agencies/officers related;  General information sharing about COVID-19/not related to police responses/actions |
| **Information gathering**  **(focal issue #3)** | Suspects related;  Missing person |
| **Police communication of administrative and mundane information**  **(focal issue #4)** | Administrative information sharing (e.g., routine police operations, recruitment and retainment, officer deployment, media advisory, instruction on crime reporting, and other neutral, general information and responses);  Police use of force, inappropriate behaviors, and citizen complaints |
| **Traffic information**  **(focal issue #5)** | Traffic related |
| **Case update**  **(focal issue #6)** | Case update without soliciting information |
| **Community engagement and outreach**  **(focal issue #7)** | Community-oriented meetings, activities, and actions (e.g., inviting community members to join police meeting and activities, sharing success of the agency, highlighting achievements of individual officers, volunteer recruitment, community survey etc.);  Interactions with communities (e.g., thanks for support and donation, holiday wishes, memorial posts, humorous tweets, save of animals, virtual ride along etc.);  Crime prevention tips, general suggestions and reminders for safety and wellness |

Exemplary tweets for the seven (7) categories:

1. Civil unrest related (e.g., “Thank you to the hundreds who made their voices heard during a peaceful demonstration downtown this afternoon/evening. No MNPD arrests during the march”).
2. COVID-19 related (e.g., “Thanks for staying home, limiting your time outdoors, & staying 6 feet apart. We're doing our part to educate communities on congregating in public spaces & practicing social distancing. Today, we visited 1,203 public places like Willowbrook Park in the confines of @NYPD121Pct”).
3. Police gathering of information (e.g., “Do you recognize this guy? He shoplifted from Crossland’s Tool Rental near 29th and S. Santa Fe. If you know who he is, call Crime Stoppers at 405/235-7300 or leave a tip online at http://okccrimetips.com. You can remain anonymous and may earn a cash reward. 2020-0009560”).
4. Police communication of administrative and mundane information (e.g., “While MPD facilities remain open at this time, we request that citizens file non-emergency police reports online: http://bit.ly/2Qw4ZcP. In an emergency, call 911. For non-emergencies & request city services, call 311. For all adjustments, please visit: <https://bit.ly/33EXAxf>”).
5. Police communication of traffic information (e.g., “#TrafficAlert All Northbound lanes of traffic on South Freeway at Felix are being shut down due to a major accident. Expect lengthy delays.”).
6. Police communication of case updates (e.g., “There will be a continuing police presence in the area as the scene investigation is conducted.”).
7. Community engagement and outreach (e.g., “#HappeningNow: @NYPDCommAffairs is in Harlem engaging with community members and enjoying some fun activities with the kids. At 6 pm, there will be a gun violence town hall to discuss how cops and the community can work together towards a safer New York”).
