# Supplementary material for: Reaching and engaging people: Analyzing tweeting practices of large U.S. police departments pre- and post- the killing of George Floyd
Source: PLoS One. 2022 Jul 14;17(7):e0269288. doi: 10.1371/journal.pone.0269288 (PMC9282545; doi:10.1371/journal.pone.0269288)
Supplement: S3 Table — (DOCX) [file pone.0269288.s003.docx]

**Table 3S**. Random forest and multiclass boosted trees classifier

- A random forest classifier generates a large number of decision trees (i.e., a map of the possible outcomes of a series of related choices) on subsamples of the dataset and uses averaging (selecting the most common output) at the end of the process to improve the predictive accuracy and control over-fitting. For further information, see <https://scikit-learn.org/stable/modules/ensemble.html#forests-of-randomized-trees>.
- A multiclass boosted trees classifier was also trained. A boosted trees classifier also combines decision trees, but the trees are built in a forward stage-wise manner and the combining process starts at the beginning, instead of at the end. For further information, see <https://www.tensorflow.org/decision_forests/api_docs/python/tfdf/keras/GradientBoostedTreesModel>.
- The random forest classifier performed better in our case. The overall accuracy and kappa value obtained from the random forest classifier were approximately 5% higher than those of the boosted trees classifier. Accuracy rates for the 7 subcategories were about the same or slightly higher when using the random forest classifier.
