# Supplementary material for: Reaching and engaging people: Analyzing tweeting practices of large U.S. police departments pre- and post- the killing of George Floyd
Source: PLoS One. 2022 Jul 14;17(7):e0269288. doi: 10.1371/journal.pone.0269288 (PMC9282545; doi:10.1371/journal.pone.0269288)
Supplement: S4 Table — (DOCX) [file pone.0269288.s004.docx]

**Table 4S**. Exemplary tweets illustrating sentence-level pleasant or attractive vs. unpleasant or aversive emotion^[[1]](#footnote-1)^*

Some tweets with the highest emotional valence scores:

- Our country is worth celebrating, but please do so responsibly.
- A very special THANK YOU to community members Lisa &amp; local group for your very generous donation of food to #SantaAnaPD officers and professional staff today! We thank you all for your continues support #takingcareofoneanother
- We love our fans, but we really love fan mail! Thank you for the kind words, @BackTheBlue_860 and for your support! #TeamRCSD
- We want to wish a #HappyBirthday to our new friend Gemma!!! 🎉🎂🎉 To celebrate Gemma turning 6, @SDSOLakeside Deputies dropped by on her special day to wish her the very best! Thanks for your support, Gemma!!! 😊 #Friendship #KeepingYouSafe #InYourCommunity
- If you’re healthy and feeling well, blood donations are very much needed right now! Please consider making an appointment at:
- We hope everyone has a very safe and happy Independence Day!
- Don’t fret mask were worn, but of course it’s really impossible to eat the delicious food at top golf with a mask on ☺️
- “Hollywood got something right, everybody loves Raymond! Raymond’s fun was pure gold, he was pure gold,” said @HCSO_D2Patrol.

Some tweets with the lowest emotional valence scores:

- @thatelliotmetz KC stats March 16-30: 2019 Total Crashes 410 Injury Crashes 98 Fatalities 3 2020 Total Crashes 419 Injury Crashes 134 Fatalities 1
- Carlos Adams 43, was arrested and charged with First Degree Murder (in Perpetration to/wit Aggravated Robbery) and Especially Aggravated Robbery.
- CORRECTION: This crash was NOT a fatal, however one driver did suffer serious bodily injury and has life threatening injuries.
- #SantaAnaPD Arrests High Risk Sex Offender
- Illegal Firearms Arrests
- Gang Member Robbery Suspects Arrested
- Assault Suspect Stabbed By Victim, Non-Life Threatening Injury
- These 122 individuals were arrested for 382 various felony crimes from serious violence, robbery, burglary, looting, pursuits, stolen vehicles, weapons, drugs etc.

1. *For tweets with more than one sentence, sentence-level emotional valence scores were averaged. Technical details about Rinker’s *sentimentr* package can be seen: <https://cran.r-project.org/web/packages/sentimentr/sentimentr.pdf> [↑](#footnote-ref-1)
