# Supplementary material for: Reaching and engaging people: Analyzing tweeting practices of large U.S. police departments pre- and post- the killing of George Floyd
Source: PLoS One. 2022 Jul 14;17(7):e0269288. doi: 10.1371/journal.pone.0269288 (PMC9282545; doi:10.1371/journal.pone.0269288)
Supplement: S5 Table — (DOCX) [file pone.0269288.s005.docx]

**Table 5S**. Supplementary details about the results of the multiclass random forest classifier

- In classification problems, accuracy indicates the number of correct predictions made by the model over all predictions made (with a range of 0 to 1), and kappa indicates the extent to which the prediction agrees with the actual value beyond what would be expected based on chance alone (with a range of -1 to +1).
- In brief, 10-fold cross-validation partitions the original training set into 10 equal size subsamples. Of the 10 subsamples, a single subsample is retained as the validation data for testing the prediction model, and the remaining 9 subsamples are used as training data. The cross-validation process is then repeated 10 times, with each of the 10 subsamples used exactly once as the validation data. The 10 results can then be averaged to produce a single estimation.
- While the classification was not perfect, our trained random forest classifier represented an important advancement from previous efforts. In a study most similar to ours, Tiry and colleagues (2019) achieved a 72% accuracy of classifying tweets from U.S. police departments and did not report by-class accuracy. See <https://www.urban.org/sites/default/files/publication/99786/social_media_guidebook_for_law_enforcement_agencies_0.pdf>
